# Characterization of the gastric mucosal microbiota in tumoral and peritumoral mucosa in patients with advanced gastric cancer from Northwest China

**DOI:** 10.3389/fmicb.2026.1763714

**Published:** 2026-06-17

**Authors:** Hongtai Cao, Qi Wang, Wen Ren, Anqi Wang, Wenji Tian, Dekui Zhang, Juanjuan Chen

**Affiliations:** 1 Department of General Surgery, The Second Hospital and Clinical Medical School, Lanzhou University, Lanzhou, Gansu, China; 2 Cuiying Biomedical Research Center, The Second Hospital and Clinical Medical School, Lanzhou University, Lanzhou, Gansu, China; 3 NHC Key Laboratory of Diagnosis and Therapy of Gastrointestinal Tumor, Gansu Provincial Hospital, Lanzhou, Gansu, China; 4 Department of Oncology, Gansu Provincial Central Hospital, Lanzhou, Gansu, China; 5 Department of Gastroenterology, Lanzhou University Second Hospital, Lanzhou, Gansu, China

**Keywords:** advanced gastric cancer, gastric mucosal microbiome, metabolic reprogramming, MetaPhlAn4 and kaiju, predictive functional pathways, tumor and peritumor

## Abstract

**Introduction:**

The gastric microbiota affects tumor development and treatment response, yet the characteristics and interactions of mucosal bacteria and fungi in advanced gastric cancer (AGC) remain unclear.

**Methods:**

Here we analyzed 177 mucosal samples (88 peritumoral and 89 tumoral) from 91 AGC patients in Northwest China using shotgun metagenomic sequencing.

**Results:**

MetaPhlAn4 and Kaiju were used to annotate the gastric mucosal microbial composition. MetaPhlAn4 has identified 12 phyla (no phylum-level differences), 98 genera and 278 species. PERMANOVA revealed age and tumor location significantly influenced microbial composition in tumoral mucosa. Wilcoxon signed-rank test revealed that 10 species including *Serratia surfactantfaciens*, *Pseudomonas protegens*, *Treponema pectinovorum*, *Streptococcus anginosus*, *Bacteroides heparinolyticus*, *Selenomonas sputigena*, and *Mogibacterium diversum* were significantly enriched in tumoral tissue, whereas five species including *Actinomyces graevenitzii*, *Gemella sanguinis*, *Porphyromonas pasteri*, *Helicobacter pylori*, and *Leptotrichia* sp. oral taxon-215 were more abundant in peritumoral mucosa. HUMAnN4 showed tumor-enriched bacteria were involved in metabolic pathways including polysaccharide degradation, biosynthesis of fatty acids, nucleotides, and arginine/histidine/purine/pyrimidine, which were primarily linked to *S. surfactantfaciens*. Peritumor-enriched bacteria were associated with L-tryptophan biosynthesis, L-arginine degradation, and TCA cycle. Kaiju annotation further revealed 2,429 bacteria, 12 archaea, 74 viruses, 82 fungi, and 63 other eukaryota species, among which the majority of significantly different species were enriched in the tumoral mocusa. Mycobiome analysis revealed eight fungal phyla, 82 genera and 82 species. PERMANOVA revealed that age had a significant effect on fungal composition in peritumoral mucosa, and five species including *Saccharomyces cerevisiae*, *Aspergillus ochraceoroseus*, *Aspergillus fumigatiaffinis*, *Mitosporidium daphniae*, and *Puccinia striiformis* were significantly positively correlated with age. Alpha diversity using Shannon index was significantly reduced in peritumoral mucosa at both genus and species levels. Wilcoxon signed-rank test revealed that all the significantly different fungi, including eight phyla, 46 genera, and 42 species were significantly enriched in tumoral mucosa. Correlation analysis indicated tumor-enriched bacteria were positively correlated with tumoral fungi but negatively with peritumoral fungi, suggesting possible synergistic bacteria-fungi interactions.

**Discussion:**

This study comprehensively characterizes the gastric mucosal bacteriome and mycobiome in AGC, illuminates potential microbiota-mediated carcinogenic mechanisms, identifies candidate biomarkers, and fills a regional research gap.

## 1 Introduction

Gastric cancer (GC) is the fifth most common cancer and the third leading cause of cancer-related deaths worldwide (Smyth et al., 2020) globally, and is diagnosed histologically after endoscopic biopsy and staged using CT, endoscopic ultrasound, PET, and laparoscopy (Bray et al., 2024). In China, the incidence and mortality rates of GC exceed global averages, with approximately 70% of patients diagnosed at locally advanced stages (II and III), known as advanced GC (AGC) (Han et al., 2024). These AGC patients face a poor prognosis due to deep tumor invasion and frequent lymph node metastasis, resulting in a 5-year overall survival rate of only 30 to 40% (Zhang et al., 2024). Therefore, there is an urgent need to develop novel prevention and treatment strategies to reduce AGC mortality.

Emerging evidence indicates that the progression of GC is closely linked to dysregulation of the gastric mucosal microenvironment (Coker et al., 2018; Yang et al., 2023; Zeng et al., 2024). The gastric mucosa, as a key barrier against pathogens and a site of metabolic activities, undergoes significant microbial shifts during the development of AGC (Dai et al., 2021). Liu et al. reported enrichment of opportunistic pathobionts (e.g., *Fusobacterium*, *Parvimonas*, *Veillonella*, *Prevotella* and *Peptostreptococcus*), and depletion of commensals (e.g., *Bifidobacterium*, *Bacillus* and *Blautia*) in GC tissues (Liu et al., 2022). They also identified eight bacterial taxa, including *Veillonella* and *Helicobacter*, as potential biomarkers for distinguishing GC from superficial gastritis (AUC = 0.85; Liu et al., 2022). The oral-gastric microbial axis may further contribute to carcinogenesis, with oral-derived microbes such as *Streptococcus anginosus* and *Prevotella melaninogenica* implicated in gastric inflammation or malignant transformation, partly through microbial metabolites like lactate, nitrite, and acetaldehyde (Xia et al., 2025). Proposed mechanisms of bacterial carcinogenesis include: (1) microbial metabolites (e.g., indole-3-propionic acid) inhibiting CD8 + T cell function and antitumor immunity (Jia et al., 2024); (2) bacteria such as *Helicobacter pylori* (Hp) and *Streptococcus* producing reactive oxygen species that damage DNA (Nakamura and Takada, 2021); (3) bacterial modulation of cytokines (e.g., VEGF) to promote angiogenesis and activate *β*-catenin to enhance cell migration (Mahaki et al., 2025; Mondal et al., 2025); (4) modulation of pro-inflammatory cytokines (e.g., TNF-*α* and IL-6) to establish a pro-tumor microenvironment (Sun et al., 2025). Additionally, microbial imbalance can reprogram tumor cell metabolism, affecting pathways such as amino acid metabolism and glycolysis to support proliferation and survival (Shang et al., 2024).

Despite these advances, most studies have focused solely on bacteria, neglecting fungal communities, an integral component of the gastric microbiome. Although fungi such as *Candida* (Dohlman et al., 2022), *Cutaneotrichosporon*, *Malassezia*, *Solicoccozyma*, and *Archaeorhizomyces* (Bimela et al., 2021; Zhang et al., 2022) are reported to be enriched in GC, their functional roles and interactions with bacteria remain poorly understood. This gap limits a holistic view of microbial contributions to AGC progression and hinder the development of microbiome-targeted therapies. Beyond bacteria and fungi, viruses including Epstein–Barr virus (Liang et al., 2014), human papillomavirus and herpesvirus, and hepatitis virus (Niedzwiedzka-Rystwej et al., 2020) have also been implicated in gastric carcinogenesis.

In summary, while the involvement of gastric microbiota in GC is established, key limitations remain. Variations in sample types, sequencing depth, and analytical methods impede cross-study comparability. Moreover, the influence of lifestyle and diet on both GC risk and microbiota composition underscores the need for region-specific studies. To address this, we used shotgun metagenomic sequencing combined with kaiju, MetaPhlAn4.0, and HUMAnN4.0 to comprehensively profile the bacterial and fungal communities, interkingdom interactions, and functional potential of the gastric mucosa in AGC patients from Northwest China. Our findings aim to provide preclinical evidence for microbiota-targeted interventions and inform novel therapeutic strategies for AGC.

## 2 Materials and methods

### 2.1 Subjects

Ninety-one AGC patients were enrolled from Lanzhou University Second Hospital from December 2021 to August 2022.

The inclusion criteria (all must be met simultaneously) were: (1) Resided in Northwest China (Shaanxi, Gansu, Ningxia, Qinghai, Xinjiang) for at least 5 years; (2) Age between 18 and 85 years old, with no gender restrictions; (3) Histopathologically confirmed primary gastric adenocarcinoma via postoperative pathology; (4) AJCC 8th edition TNM staging is stage II-IV; (5) Those patients with AGC are not contraindicated for surgery; (6) Available samples of the primary lesion and paired adjacent mucosa (≥5 cm from the tumor margin); (7) First diagnosis without prior clinical treatment including chemotherapy, radiotherapy, targeted therapy, immunotherapy and no neoadjuvant treatment; (8) Signed written informed consent and agreement to provide gastric mucosa samples and clinical data; (9) Stable dietary habits over the past 6 months with no major changes in dietary structure.

The exclusion criteria (any one led to exclusion) were: (1) Prior receipt of any gastric cancer-related anti-tumor therapy; (2) Use of proton pump inhibitors within 4 weeks; (3) Use of antibiotics, probiotics, glucocorticoids, non-steroidal anti-inflammatory drugs (NSAIDs), or immunosuppressants within 2 months before surgery; (4) Presence of other malignant tumors, severe dysfunction of the heart, liver, kidney or lung/liver cirrhosis, chronic renal failure, infections (HBV/HCV positive, tuberculosis, or undergoing *Helicobacter pylori* eradication therapy), or other gastrointestinal diseases (e.g., inflammatory bowel disease, active peptic ulcer); (5) Special physiological conditions such as pregnancy, lactation or severe malnutrition (BMI < 18.5); (6) Inability to obtain qualified mucosal samples, incomplete clinical or pathological data, or inability to cooperate with sampling or follow-up; (7) Long-term heavy alcohol consumption (> 40 g/day), drug abuse, or inability to cooperate due to mental illness; (8) Body mass index (BMI) over 30.

### 2.2 Phenotypes and samples collection

Prior to gastrectomy, comprehensive basic clinical data and relevant information for all AGC patients were systematically collected, ensuring a holistic dataset for subsequent analysis (Supplementary Table S1).

Gastric mucosal samples were carefully collected from the resected tumor and adjacent tumor tissues (at least 5 centimeters distant from the tumor margin) using a sterile surgical knife. The scraped mucus specimens were promptly transferred into sterile 1.5 mL tubes and stored at −80 °C for subsequent DNA extraction and shotgun metagenomic sequencing. Meanwhile, negative control samples were collected by swabbing the surface of the operating table and the outer surface of doctors’ masks (*n* = 2 per location). After the sample collection was finished, all the samples were transported to the laboratory by dry ice for following DNA extraction and shotgun metagenomic sequencing.

### 2.3 Shotgun metagenomic sequencing and analysis

#### 2.3.1 DNA extraction and quality control

The frozen gastric mucosal samples were thawed on ice, and total DNA was extracted using the phenol/trichloromethane method. To eliminate RNA contamination, the extracts were treated with DNase-free RNase. The quantity and quality of DNA were evaluated using a NanoDrop spectrophotometer, 1.5% agarose gel electrophoresis, and Qubit. Only DNA samples meet all the following criteria were selected for subsequent library construction: (1) A260/A280 ratio between 1.8 and 2.0; (2) intact main bands without noticeable smearing on the gel, along with a clear single high-molecular-weight band above 15 kb; (3) double-stranded DNA concentration ≥10 ng/μL and total DNA amount >100 ng. The DNA samples were classified as A, B, C, or D based on their contents and integrity, with A and B being directly usable for library construction and sequencing. Notably, all the samples except for negative controls (*n* = 4) fell under classification A, indicating high-quality DNA.

The MGIEasy Universal DNA Library Preparation Reagent Kit was employed to construct DNA library. Paired-end (PE) shotgun metagenomic sequencing was conducted on the DNBSEQ T7 platform with an insert size of 350 bp and a read length of 150 bp.

#### 2.3.2 Obtaining high-quality reads

Raw sequencing reads were filtered to remove low-quality reads using Fastp with default parameters. Specifically, reads were discarded if they met any of the following criteria: an average quality score below Q20, ambiguous bases (N content > 1%), or a length shorter than 50 bp. Only reads with final length ≥ 50 bp, N content ≤ 1%, and an average Q30 > 85% were retained. Subsequently, human DNA reads were eliminated using Bowtie 2 with default settings (average host read content: 96.97%). The remaining reads, defined as high-quality microbial reads (averaging 4.099 Gb per sample), were utilized for taxonomic profiling and prediction of functional pathways (Supplementary Table S3).

#### 2.3.3 Bacterial taxonomic and functional profiling acquisition

We employed MetaPhlAn4.0 to characterize the microbial community composition derived from metagenomic shotgun sequencing data (Supplementary Table S5). Subsequently, the bacterial phyla, genera and species were extracted and analyzed (Supplementary Table S6–S8).

To efficiently and precisely profiles the abundance of microbial metabolic pathways and other molecular functions from metagenomic sequencing data, we utilized HUMAnN4.0. The UniRef pathways and GO terms were obtained (Supplementary Tables S11–S13).

#### 2.3.4 Diversity calculation

For beta-diversity analysis, we utilized the diversity function from the vegan package in R 4.0.3, calculating the Bray-Curtis distance at both the genus and species levels, and the results were visualized as PCoA plots.

To evaluate alpha diversity, we computed the Shannon, Simpson and Inverse Simpson indexes using R software, providing measures of within-sample species richness and evenness.

#### 2.3.5 Kaiju annotation

To further know the overall changes except for bacteria, kaiju classifer was used to obtaning viral, fungal, and bacterial profiles from the high-quality metagenomic sequencing reads. Kaiju is a highly efficient tool renowned for its speed and sensitivity in taxonomic classification of metagenomic data, following the protocol described by Menzel et al. (2016) (kaiju -t nodes.dmp -f kaiju_db.fmi -i inputfile.fastq -o fungi.taxa). The mycobial profile were included in Supplementary Table S3, and the phylum, genus and species profile were extracted and analyzed in Supplementary Table S4.

### 2.4 Statistical analysis

Rarefaction curves were used to evaluate the sufficiency of the sequencing depth by randomly selecting eight samples, subsampled the sequencing data from 1 to 100% of the original read depth, performed species annotation and diversity index calculations on each subsampled dataset, and plotted the relationship between sampling depth and observed diversity.

*Decontam* package in R (version 4.5.3) was used to identify and remove possible contaminants from sequencing data to improve the accuracy and quality of the data according to Benjamin Callahan and Nicole Davis (Introduction to *decontam*).

To assure the accuracy of the subsequent taxonomic profiling, taxa present in fewer than 5% of all samples (i.e., appearing in less than 5 out of 100 samples) were removed according to Zhu et al. (doi:10.1038/s41467-020-15457-9). This step excluded rare biosphere members potentially arising from sequencing errors, cross-contamination, or PCR artifacts. Additionally, taxa with an alpha diversity (Shannon index) value below 0.1 were also discarded (Supplementary Table S5C).

PERMANOVA was used to explore the impact of clinical phenotypes on mucosal microbiota. Wilcoxon signed-rank test and Wilcoxon rank-sum test was employed to compare the differences of the abundances of fungi and bacteria between tumoral and peritumoral mucosa. Kruskal-Wallis test was applied to analyze the variations among different tumorigenic sites. Spearman’s rank correlation analysis was conducted to explore the relationships between gastric mucosal bacteria and fungi across all samples.

In all statistical analyses, *p* < 0.05 was considered statistically significant.

## 3 Results

### 3.1 Phenotypic analysis of the patients with AGC

A total of 91 AGC patients underwent surgical resection at Lanzhou University Second Hospital were included. Clinical staging of the patient cohort (female: male = 13:78, mean age: 58.84 ± 9.19 years, mean weight: 64.46 ± 9.40 kg, mean BMI: 22.65 ± 2.84) showed that 25 patients were in Stage II, 33 in Stage III, and 33 in Stage IV. For surgery, 41 patients underwent distal gastrectomy, 42 had total gastrectomy, and eight received proximal gastrectomy. Chemotherapy was administered to 55 patients, while the other 36 did not receive it. Regarding tumor locations, 52 cases involved the gastric antrum and angle, 13 were located in the gastric body, and 26 were in the cardia and fundus (Supplementary Table S1).

Finally, 88 peritumoral and 89 tumoral mucosa were collected for shotgun metagenomic sequencing and analysis and the total DNA were extracted and quality controlled (Supplementary Table S2). An average of 4,099 million bases (high-quality reads, 4.099 Gb) were obtained for each sample to perform subsequent taxonomic and functional annotation and analysis (Supplementary Table S3). *Decontam* statistical analysis revealed that there is no significant correlation between library size and typical contaminant taxa based on DNA concentration of all samples (Supplementary Table S4).

### 3.2 Bacterial differences between tumoral and peritumoral mucosa

To accurately characterize the gastric mucosal bacterial communities, Metaphlan4.0 was employed (Supplementary Table S5A). The flattened rarefaction curves confirmed that the sequencing depth is enough for taxonomic analysis (Supplementary Figure S1; Supplementary Table S5B). To make the results of bacteria more credible, we have filtered the samples with Shannon index less than 0.1, and finally 73 tumoral and 60 peritumoral mucosal samples were used for differential analysis (Supplementary Tables S5C,D), in which 56 samples were paired (tumoral and peritumoral samples from the same patient). Wilcoxon signed-rank test and Wilcoxon rank-sum test were used for 56 paired samples and 133 samples, respectively.

**Figure 1:**
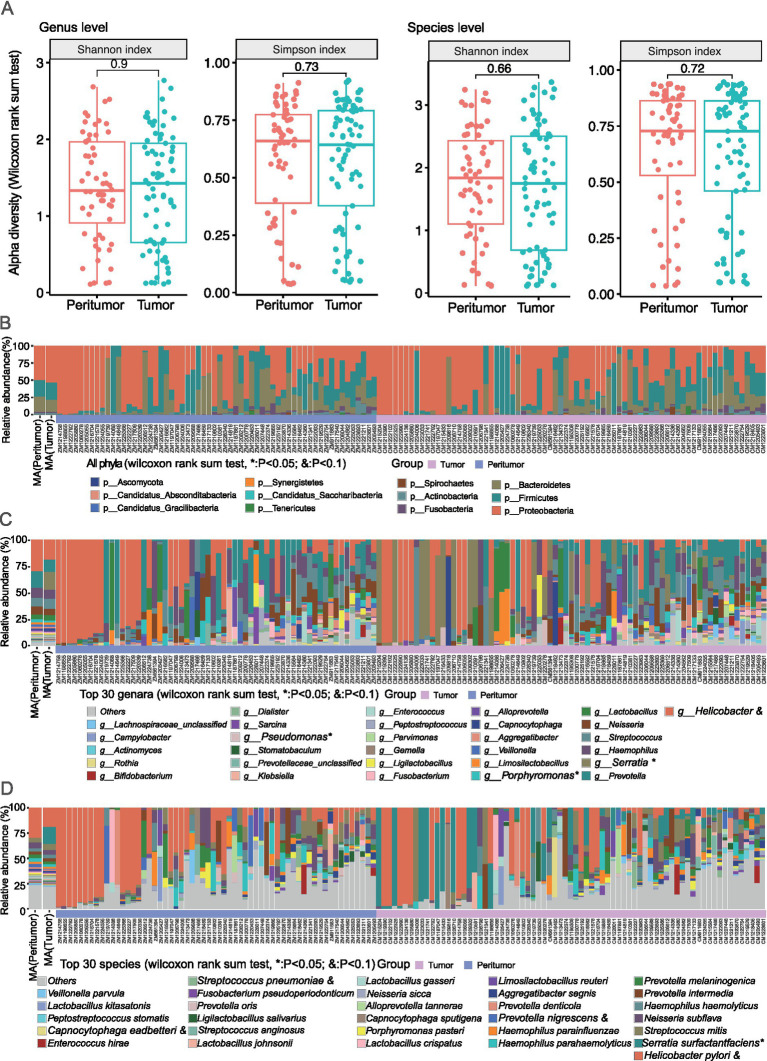
Alpha diversity and comparison of the top abundant bacteria between tumoral and peritumoral mucosa. **(A)** Alpha diversity of the bacterial genera and species calculated using Shannon, Simpson, and Inverse Simpson indecies. **(B)** Phylum-level composition. **(C)** The top 30 most abundant genera. **(D)** The top 30 most abundant species. * indicates genera and species with significant differences between tumoral and peritumoral mucosa.

**Figure 2:**
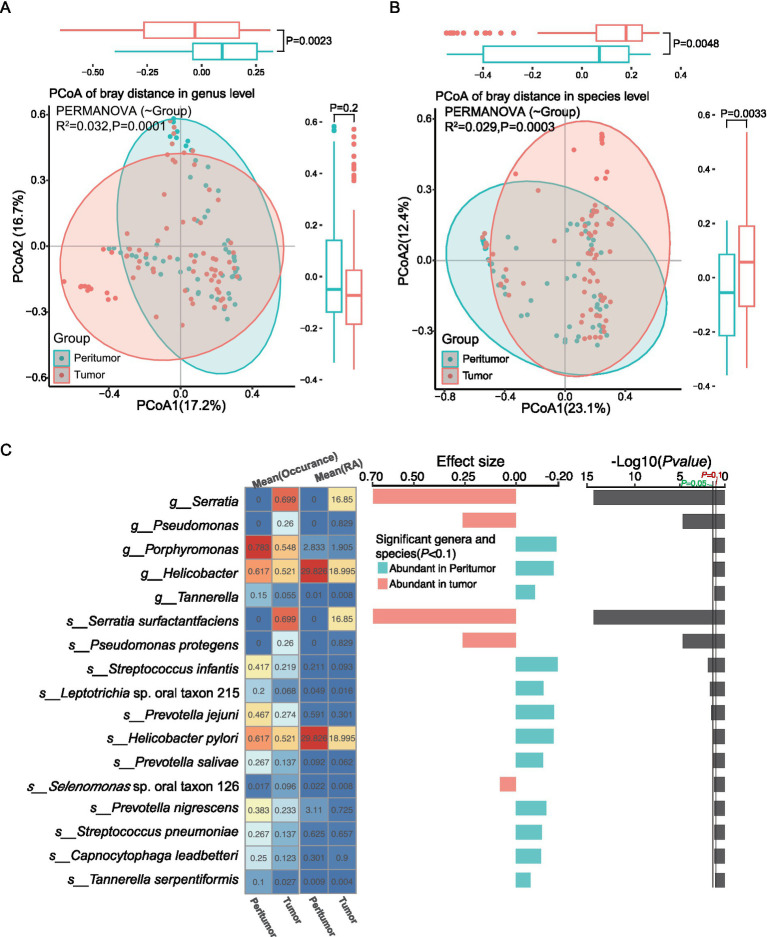
Characterization of the gastric mucosa bacterial microbiota annoated by MetaPhlAn4. Principal coordinate analysis (PCoA) revealed significant differences in bacterial genera **(A)** and species **(B)** between tumor and peritumoral mucosal samples. **(C)** Significantly differentially abundant genera and species revealed by Wilcoxon signed-rank test.

Alpha diversity calculation using Shannon and Simpson indices revealed no significant differences between tumoral and peritumoral samples (Figure 1A). However, PCoA based on Bray-Curtis distances demonstrated significant differences between tumoral and peritumoral mucosa at both the genus and species levels (Figure 2A, genus level, *R*^2^ = 0.032, *p* = 0.0001; Figure 2B, species level, *R*^2^ = 0.029, *p* = 0.0003).

Eleven bacterial phyla were identified, with Proteobacteria, Firmicutes, Bacteroidetes, Fusobacteria, and Actinobacteria comprising the five most abundant phyla (Supplementary Table S5E; Figure 1B). Wilcoxon signed-rank test identified three significantly different phyla between tumor and peritumor mucosa, with Spirochaetes and Firmicutes enriched in tumor while Proteobacteria in peri-tumor. Wilcoxon rank-sum test identified no significant differences between tumoral and peritumoral mucosa in these phyla (Supplementary Table S5E).

At the genus level, 96 genera were annotated. Wilcoxon signed-rank test identified nine significantly different genera, with *Actinomyces* and *Helicobacter* enriched in peritumoral mucosa while *Serratia*, *Olsenella*, *Bacteroides*, *Slackia*, *Lachnospiraceae_unclassified*, *Treponema*, and *Pseudomonas* were enriched in tumoral mucosa (Figure 2C; Supplementary Table S5F). Wilcoxon rank-sum test identified three significantly different genera, with *Serratia* and *Pseudomonas* enrichd in tumoral mucosa, whereas *Porphyromonas* enriched in peritumoral mucosa (Supplementary Table S5F). Analysis of the most abundant genera revealed that high-abundant taxa including *Serratia* was preferentially enriched in tumoral mucosa, while *Porphyromonas* and *Helicobacter* exhibited greater abundance in peritumoral mucosa, other high-abundance genera including *Prevotella*, *Haemophilus*, *Streptococcus*, *Neisseria*, *Lactobacillus*, *Limosilactobacillus*, *Veillonella* showed no significant differences between two sites (Figure 1C; Supplementary Table S5F).

Totally 278 bacterial species were annotated. Wilcoxon signed-rank test identified 15 significantly different species, with *Serratia surfactantfaciens*, *Treponema pectinovorum*, *Streptococcus anginosus*, *Mogibacterium diversum*, *Lachnospiraceae* bacterium_oral_taxon_096, *Bacteroides heparinolyticus*, *Slackia exigua*, *Selenomonas sputigena*, *Pseudomonas protegens*, and *Ligilactobacillus salivarius* enriched in tumoral tissues, whereas *Helicobacter pylori*, *Porphyromonas pasteri*, *Gemella sanguinis*, *Actinomyces graevenitzii*, and *Leptotrichia* sp. Oral_taxon_215 enriched in peritumoral tissues. Wilcoxon rank-sum test identified six significantly different species, with *Serratia surfactantfaciens*, *Pseudomonas protegens*, and *Treponema pectinovorum* significantly abundant in tumoral mucosa, whereas *Streptococcus infantis*, *Leptotrichia* sp. oral taxon_215, *Prevotella jejuni* were enriched in peritumoral mucosa (Figure 2C). Analysis of the top abundant species showed that *Serratia surfactantfaciens*, *Streptococcus anginosus,* and *Ligilactobacillus salivarius* were preferentially enriched in tumoral mucosa, while *Helicobacter pylori* and *Porphyromonas pasteri* displayed higher abundances in peritumoral mucosa (Supplementary Table S5G; Figure 1D).

### 3.3 PERMANOVA of phenotypes on gastric bacteria in tumoral and peritumoral mucosa

PERMANOVA revealed that age and tumor locations had significant impacts on gastric mucosal species and genera in tumoral mucosa, respectively (Table 1). We subsequently analyzed age-related bacteria (Supplementary Table S5H) and compared bacterial compositional differences across three different cancerous sites (the antrum and angle, corpus, and cardia-fundus regions; Supplementary Table S5I).

**Table 1:** PERMANOVA of phenotypes on bacterial genera, species, and pathways in tumor and peritumor mucosa.

Phenotypes	Tumoral mucosa						Peritumoral mucosa					
	Genus level		Species level		Pathway level		Genus level		Species level		Pathway level	
PERMANOVA test	*R* ^2^	*p* value	*R* ^2^	*p* value	*R* ^2^	*p* value	*R* ^2^	*p* value	*R* ^2^	*p* value	*R* ^2^	*p* value
Gender	0.019	0.177	0.023	0.062	0.002	0.877	0.024	0.182	0.021	0.217	0.023	0.249
Age	0.021	0.117	0.026	**0.034**	0.005	0.663	0.015	0.509	0.019	0.312	0.022	0.255
Height	0.011	0.615	0.013	0.496	0.000	0.979	0.021	0.214	0.020	0.253	0.015	0.405
Weight	0.014	0.47	0.014	0.363	0.013	0.365	0.019	0.295	0.020	0.269	0.018	0.359
BMI	0.018	0.229	0.015	0.359	0.017	0.262	0.026	0.095	0.028	0.078	0.031	0.137
Chemotherapy	0.010	0.762	0.010	0.792	0.001	0.963	0.012	0.732	0.013	0.724	0.004	0.823
Tumor location	0.044	**0.044**	0.032	0.3	0.008	0.883	0.048	0.094	0.032	0.496	0.048	0.197
Differentiated degree	0.034	0.763	0.030	0.91	0.025	0.708	0.047	0.585	0.057	0.271	0.025	0.878
Lauren types	0.038	0.605	0.036	0.692	0.030	0.597	0.041	0.794	0.040	0.847	0.025	0.876
Tumor stage	0.030	0.316	0.024	0.593	0.016	0.647	0.043	0.173	0.032	0.515	0.037	0.347
Surgical resection method	0.030	0.322	0.029	0.386	0.023	0.452	0.027	0.722	0.030	0.582	0.025	0.598

**Figure 3:**
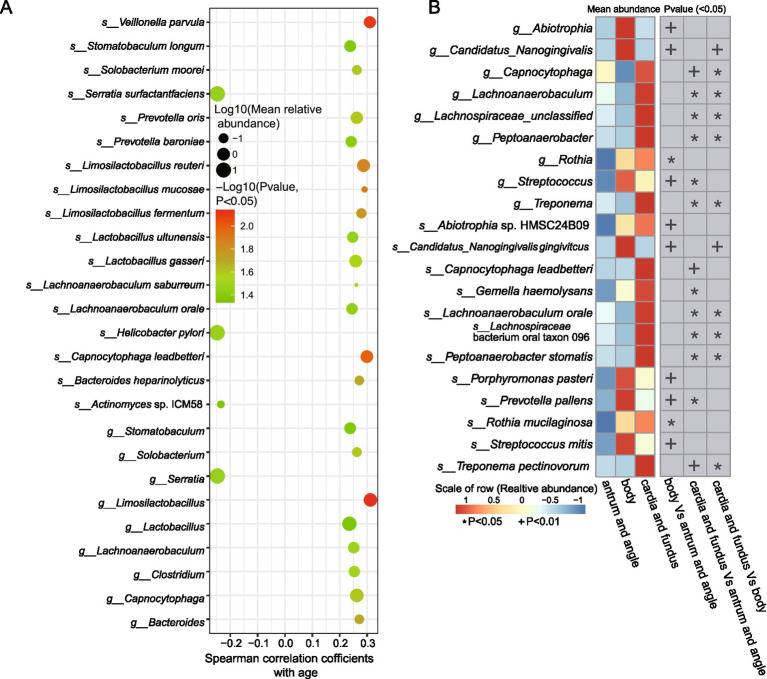
Bacterial taxa significantly associated with age and tumor location. **(A)** Bacterial genera and species significantly correlated with age in tumoral mucosa. **(B)** Bacterial genera and species showing significant differences across the three tumor locations in tumoral mucosa. s__: species; g__: genera.

For age-related species, 17 taxa were identified, of which 14 species (*Bacteroides heparinolyticus*, *Capnocytophaga leadbetteri*, *Lachnoanaerobaculum orale*, *Lachnoanaerobaculum saburreum*, *Lactobacillus gasseri*, *Lactobacillus ultunensis*, *Limosilactobacillus fermentum*, *Limosilactobacillus mucosae*, *Limosilactobacillus reuteri*, *Prevotella baroniae*, *Prevotella oris*, *Solobacterium moorei*, *Stomatobaculum longum*, *Veillonella parvula*) showed positive correlations with age. Conversely, three species including *Serratia surfactantfaciens*, *Helicobacter pylori*, and *Actinomyces* sp. ICM58 were negatively correlated with age (Figure 3A).

No significant differences in alpha diversity were observed among tumor locations (Supplementary Figure S2), whereas significant compositional differences of several genera nad species were detected across three locations (Supplementary Figure S3). Totally nine genera and 12 species were differentially distributed across the three cancerous sites (Figure 3B). Compared with the antrum and angle, the corpus exhibited significant enrichment of four genera (*Abiotrophia*, *Candidatus Nanogingivalis*, *Rothia*, *Streptococcus*) and six species (*Abiotrophia* sp. HMSC24B09, *Candidatus Nanogingivalis Gingivitcus*, *Porphyromonas pasteri*, *Prevotella pallens*, *Rothia mucilaginosa*, *Streptococcus mitis*). The cardia-fundus region displayed higher abundances of six genera (*Capnocytophaga*, *Lachnoanaerobaculum*, *Lachnospiraceae_unclassified*, *Peptoanaerobacter*, *Streptococcus*, *Treponema*) and seven species (*Capnocytophaga leadbetteri*, *Gemella haemolysans*, *Lachnoanaerobaculum orale*, Lachnospiraceae bacterium oral taxon 096, *Peptoanaerobacter stomatis*, *Prevotella pallens*, *Treponema pectinovorum*) when compared to the antrum and angle. When contrasted with the corpus, the cardia-fundus region showed significant increases in five genera (*Capnocytophaga*, *Lachnoanaerobaculum*, *Peptoanaerobacter*, *Treponema*, *Lachnospiraceae_unclassified*) and four species (*Lachnoanaerobaculum orale*, Lachnospiraceae bacterium oral taxon-096, *Peptoanaerobacter stomatis*, *Treponema pectinovorum*), while two species (*Candidatus Nanogingivalis* and *Candidatus Nanogingivalis-Gingivitcus*) were significantly decreased.

### 3.4 Differential analysis of bacterial metabolic pathways between tumoral and peritumoral mucosa

**Figure 4:**
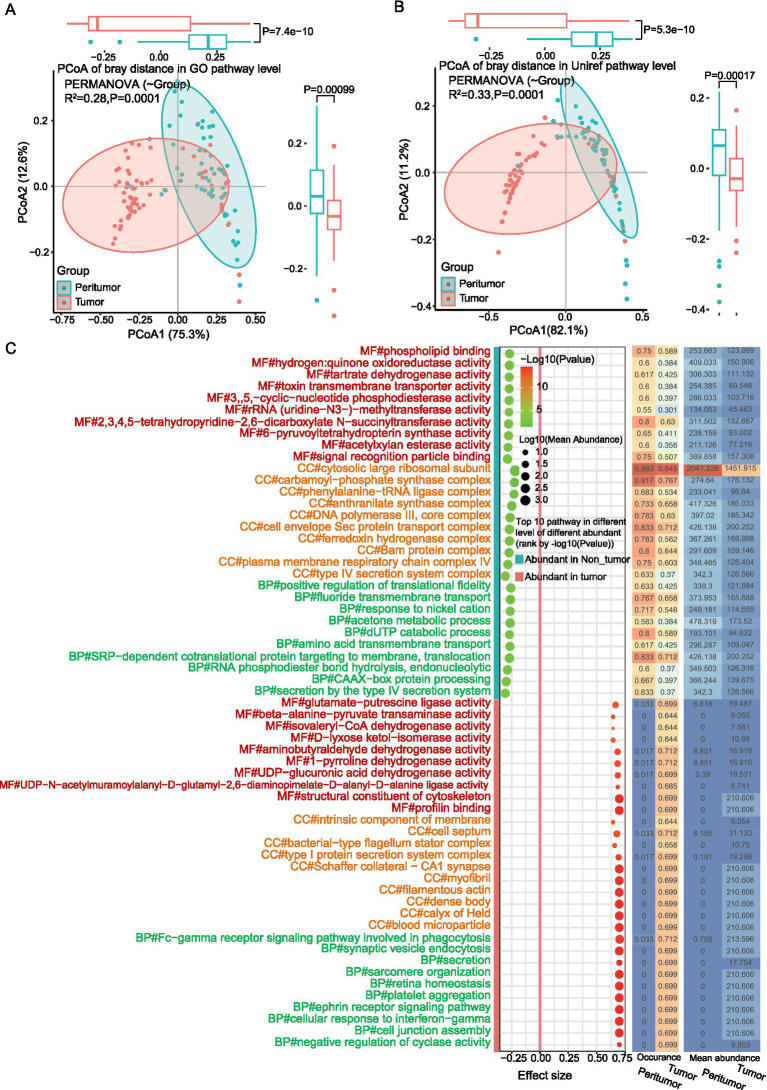
Principal coordinate analysis (PCoA) of GO terms **(A)** and UniRef pathways **(B)** between tumor and peritumoral mucosa. **(C)** GO annotations of the top 10 abundant cellular component **(CC)**, molecular function (MF), and biological process (BP) for tumoral and peritumoral mucosa, respectively.

To further characterize the gastric mucosal bacterial function, we utilized HUMAnN 4.0. PCoA based on Bray–Curtis dissimilarity revealed significant clustering differences in functional pathways between tumoral and peritumoral mucosa at both GO terms (Figure 4A; *R*^2^ = 0.28, *p* = 0.0001) and UniRef pathways (Figure 4B; *R*^2^ = 0.33, *p* = 0.0001).

#### 3.4.1 GO enrichment analysis

In total, 3,774 GO pathways were identified, encompassing 1,405 biological processes (BP), 2043 molecular functions (MF), and 326 cellular components (CC). Differential analysis revealed 1,105 significantly altered GO pathways (*p* < 0.05), including 461 BP, 573 MF, and 97 CC. Among these, 707 GO terms (306 BP, 356 MF, 45 CC) were significantly enriched in tumoral mucosa, while 397 pathways (155 BP, 217 MF, 25 CC) showed preferential enrichment in peritumoral mucosa.

In tumoral mucosa, top-enriched CC included intrinsic component of membrane and bacterial-type flagellum stator complex, with MF such as glutamate-putrescine ligase activity and structural constituent of cytoskeleton. Biological processes centered on Fc-gamma receptor signaling in phagocytosis and synaptic vesicle endocytosis (Figure 4C).

Peritumoral mucosa featured enriched CC like cytosolic large ribosomal subunit and DNA polymerase III core complex, with molecular functions including phospholipid binding and hydrogen:quinone oxidoreductase activity. Key BP involved positive regulation of translational fidelity and amino acid transmembrane transport (Figure 4C). These findings highlight distinct functional profiles between tumoral and peritumoral mucosa, linking microbial activities to cellular machinery and metabolic pathways critical for cancer progression.

#### 3.4.2 Bacterial metabolic pathways analysis

**Figure 5:**
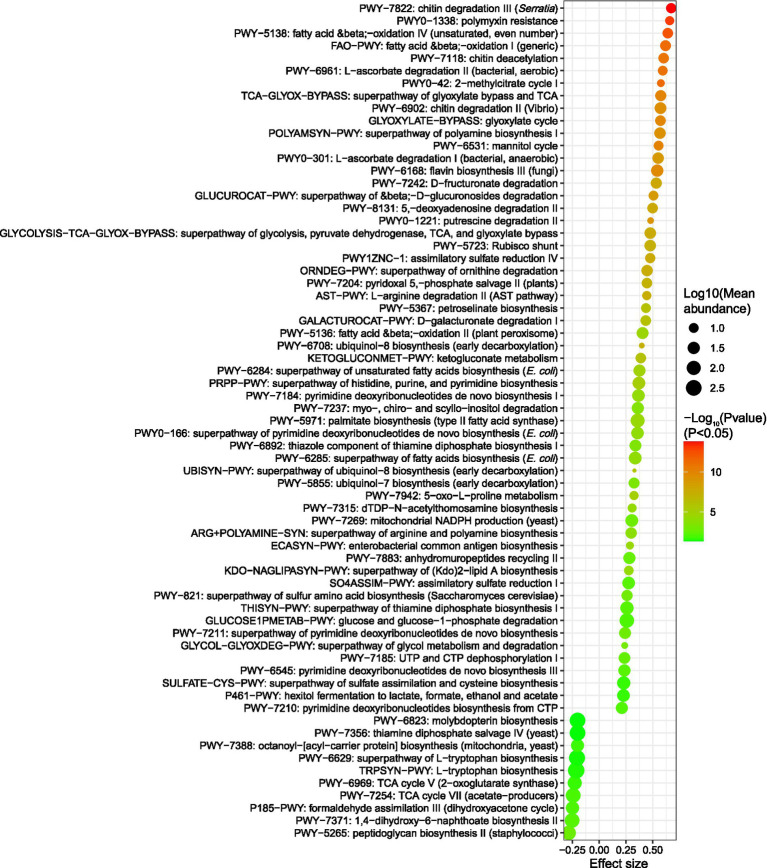
Differentially abundant UniRef pathways between tumoral and peritumoral mucosal microbiota (|Effect Size (ES)| > 0.25), indicating enrichment in either tumoral (ES > 0.25) or peritumoral (ES < -0.25) microenvironments.

A total of 342 UniRef pathways were identified across all gastric mucosal bacterial communities (Supplementary Table S5J). Among these, 76 pathways exhibited significant differences between tumoral and peritumoral mucosa (Figure 5). Notably, 50 pathways were significantly enriched in tumor-associated mucosal bacteria, including chitin degradation, polymyxin resistance, fatty acid *β*-oxidation IV/I, the glyoxylate bypass and TCA superpathway, the glycolysis/pyruvate dehydrogenase/glyoxylate bypass superpathway, the unsaturated fatty acid biosynthesis superpathway, and the *de novo* biosynthesis of pyrimidine deoxyribonucleotides superpathway, among others. Intriguingly, all these highly activated pathways were associated with *Serratia surfactantfaciens*, which was significantly enriched in the gastric tumoral mucosa of AGC patients.

Conversely, 20 pathways were predominantly enriched in peritumoral mucosal bacteria, including L-arginine degradation II, fatty acid β-oxidation II, myo−/chiro−/scyllo-inositol degradation, assimilatory sulfate reduction I, pyrimidine deoxyribonucleotides de novo biosynthesis III, and the sulfate assimilation and cysteine biosynthesis superpathway, among others (Figure 5). Notably, besides *Serratia marcescens*, *Haemophilus parahaemolyticus*, *Haemophilus parainfluenzae*, and *Pseudomonas protegens* were the key bacterial species involved in these pathways, with *Pseudomonas protegens* being enriched in tumoral mucosa (Supplementary Table S5K).

#### 3.4.3 Networks between significantly different bacteria and metabolic pathways

Further analysis focused on UniRef pathways associated with significantly differentially abundant bacteria between tumoral and peritumoral mucosa. Tumor-enriched species (*Serratia surfactantfaciens* and *Treponema pectinovorum*) and peritumoral-enriched *Leptotrichia* sp. oral_taxon_215 were not associated with any UniRef pathways. Tumor-enriched *Pseudomonas protegens* was involved in myo-, chiro-, and scyllo-inositol degradation. Peritumoral mucosal *Prevotella jejuni* participated in 20 UniRef pathways, including glycogen biosynthesis I, peptidoglycan biosynthesis I, inosine 5’-phosphate degradation, peptidoglycan biosynthesis III (mycobacteria), UDP-N-acetylmuramoyl-pentapeptide biosynthesis I/III, and adenine/adenosine salvage III. Peritumoral *Streptococcus infantis* was primarily engaged in carbohydrate, nucleotide, and aromatic amino acid metabolism, including chorismate biosynthesis I, the aromatic amino acid biosynthesis superpathway, L-isoleucine biosynthesis I, L-methionine biosynthesis II, L-cysteine biosynthesis VI, sucrose biosynthesis II, glycogen biosynthesis I/II, lactose/galactose degradation I, pentose phosphate pathway I/II, pyruvate fermentation to acetate and (S)-lactate I/lactate II, glycolysis IV, 5-aminoimidazole ribonucleotide biosynthesis I/II, inosine-5’-phosphate biosynthesis I/II, the adenosine/guanosine nucleotides *de novo* biosynthesis superpathway I/II, and adenosine/guanosine deoxyribonucleotides de novo biosynthesis II, among others (Table 2). Notably, except for PWY-7237 (myo−/chiro−/scyllo-inositol degradation) enriched in peritumoral mucosa, all other UniRef pathways associated with differentially abundant species showed no significant differences between tumoral and peritumoral mucosa, implying the potential importance of other microbial components (e.g., fungi) in this context (Supplementary Table S5K).

**Table 2:** Uniref pathways associated with significantly different bacteria between the tumoral and peritumoral mucosa.

Uniref pathways and bacteria interaction networks	*p*-value	Effect size	Mean Ab. in tumoral mucosa	Mean Ab. in peritumoral mucosa	Enrichment
Myo-, chiro- and scyllo-inositol degradation|*Pseudomonas_protegens*	0.000	0.192	0	0.668	Peritumor < Tumor
Glycogen biosynthesis I (from ADP-D-Glucose)|*Prevotella_jejuni*	0.016	−0.144	1.176	0.643	Tumor < Peritumor
Peptidoglycan biosynthesis I (meso-diaminopimelate containing)|*Prevotella_jejuni*	0.021	−0.145	1.622	0.944	Tumor < Peritumor
Inosine 5,-phosphate degradation|*Prevotella_jejuni*	0.004	−0.196	1.865	1.006	Tumor < Peritumor
Peptidoglycan biosynthesis III (mycobacteria)|*Prevotella_jejuni*	0.022	−0.144	1.667	0.987	Tumor < Peritumor
UDP-N-acetylmuramoyl-pentapeptide biosynthesis I (meso-diaminopimelate containing)|*Prevotella_jejuni*	0.038	−0.133	1.607	0.982	Tumor < Peritumor
Adenine and adenosine salvage III|*Prevotella_jejuni*	0.005	−0.155	1.216	0.765	Tumor < Peritumor
UDP-N-acetylmuramoyl-pentapeptide biosynthesis III (meso-diaminopimelate containing)|*Prevotella_jejuni*	0.031	−0.126	1.295	0.811	Tumor < Peritumor
Chorismate biosynthesis I|*Streptococcus_infantis*	0.010	−0.199	4.307	2.005	Tumor < Peritumor
Superpathway of aromatic amino acid biosynthesis|*Streptococcus_infantis*	0.009	−0.178	3.831	1.653	Tumor < Peritumor
Glycogen biosynthesis I (from ADP-D-Glucose)|*Streptococcus_infantis*	0.004	−0.235	5.294	2.225	Tumor < Peritumor
L-isoleucine biosynthesis I (from threonine)|*Streptococcus_infantis*	0.025	−0.177	4.866	2.251	Tumor < Peritumor
Lactose and galactose degradation I|*Streptococcus_infantis*	0.010	−0.216	6.575	3.089	Tumor < Peritumor
Pentose phosphate pathway (non-oxidative branch) I|*Streptococcus_infantis*	0.037	−0.148	2.884	1.274	Tumor < Peritumor
Acetylene degradation (anaerobic)|*Streptococcus_infantis*	0.017	−0.192	5.777	2.807	Tumor < Peritumor
Pyruvate fermentation to acetate and (S)-lactate I|*Streptococcus_infantis*	0.015	−0.196	4.638	2.040	Tumor < Peritumor
ppGpp metabolism|*Streptococcus_infantis*	0.046	−0.114	1.464	0.717	Tumor < Peritumor
Purine ribonucleosides degradation|*Streptococcus_infantis*	0.020	−0.189	5.829	2.753	Tumor < Peritumor
Ethanolamine utilization|*Streptococcus_infantis*	0.017	−0.192	5.777	2.807	Tumor < Peritumor
Peptidoglycan maturation (meso-diaminopimelate containing)|*Streptococcus_infantis*	0.010	−0.214	4.380	2.145	Tumor < Peritumor
Glycolysis IV|*Streptococcus_infantis*	0.022	−0.182	6.402	2.833	Tumor < Peritumor
Pyruvate fermentation to acetate and lactate II|*Streptococcus_infantis*	0.015	−0.196	4.638	2.040	Tumor < Peritumor
L-isoleucine biosynthesis III|*Streptococcus_infantis*	0.025	−0.177	4.433	2.030	Tumor < Peritumor
Sucrose degradation IV (sucrose phosphorylase)|*Streptococcus_infantis*	0.009	−0.212	4.148	3.000	Tumor < Peritumor
Glycogen degradation II|*Streptococcus_infantis*	0.005	−0.231	6.730	3.123	Tumor < Peritumor
5-aminoimidazole ribonucleotide biosynthesis I|*Streptococcus_infantis*	0.041	−0.165	4.065	2.283	Tumor < Peritumor
5-aminoimidazole ribonucleotide biosynthesis II|*Streptococcus_infantis*	0.044	−0.163	4.197	2.338	Tumor < Peritumor
inosine-5,-phosphate biosynthesis I|*Streptococcus_infantis*	0.025	−0.184	3.730	2.003	Tumor < Peritumor
Inosine-5,-phosphate biosynthesis II|*Streptococcus_infantis*	0.037	−0.170	3.776	2.016	Tumor < Peritumor
Superpathway of guanosine nucleotides de novo biosynthesis II |*Streptococcus_infantis*	0.049	−0.113	1.529	0.786	Tumor < Peritumor
Superpathway of adenosine nucleotides de novo biosynthesis II |*Streptococcus_infantis*	0.010	−0.210	3.798	2.002	Tumor < Peritumor
S-adenosyl-L-methionine salvage I|*Streptococcus_infantis*	0.044	−0.162	4.969	2.818	Tumor < Peritumor
Chorismate biosynthesis from 3-dehydroquinate|*Streptococcus_infantis*	0.006	−0.221	4.553	2.058	Tumor < Peritumor
Superpathway of 5-aminoimidazole ribonucleotide biosynthesis |*Streptococcus_infantis*	0.044	−0.163	4.197	2.338	Tumor < Peritumor
D-galactose degradation I (Leloir pathway)|*Streptococcus_infantis*	0.018	−0.196	4.970	2.360	Tumor < Peritumor
Superpathway of L-phenylalanine biosynthesis|*Streptococcus_infantis*	0.037	−0.134	2.396	1.188	Tumor < Peritumor
Queuosine biosynthesis I (de novo)|*Streptococcus_infantis*	0.016	−0.195	4.336	1.710	Tumor < Peritumor
Seleno-amino acid biosynthesis (plants)|*Streptococcus_infantis*	0.031	−0.156	3.014	0.963	Tumor < Peritumor
L-methionine biosynthesis II|*Streptococcus_infantis*	0.000	−0.218	3.686	0.727	Tumor < Peritumor
Pyruvate fermentation to isobutanol (engineered)|*Streptococcus_infantis*	0.022	−0.185	5.153	2.273	Tumor < Peritumor
Adenosine deoxyribonucleotides de novo biosynthesis II |*Streptococcus_infantis*	0.020	−0.195	3.407	1.796	Tumor < Peritumor
Guanosine ribonucleotides de novo biosynthesis|*Streptococcus_infantis*	0.041	−0.168	4.853	2.744	Tumor < Peritumor
Guanosine deoxyribonucleotides de novo biosynthesis II |*Streptococcus_infantis*	0.020	−0.195	3.407	1.796	Tumor < Peritumor
Superpathway of adenosine nucleotides de novo biosynthesis I |*Streptococcus_infantis*	0.011	−0.208	4.277	2.227	Tumor < Peritumor
Sucrose biosynthesis II|*Streptococcus_infantis*	0.011	−0.212	9.100	4.411	Tumor < Peritumor
UMP biosynthesis II|*Streptococcus_infantis*	0.038	−0.159	4.237	1.826	Tumor < Peritumor
Pentose phosphate pathway (non-oxidative branch) II |*Streptococcus_infantis*	0.035	−0.150	3.290	1.208	Tumor < Peritumor
L-cysteine biosynthesis VI (from L-methionine)|Streptococcus_infantis	0.002	−0.168	2.403	0.654	Tumor < Peritumor
tRNA charging|*Streptococcus_infantis*	0.007	−0.208	4.401	2.076	Tumor < Peritumor
UDP-N-acetyl-D-glucosamine biosynthesis I|*Streptococcus_infantis*	0.005	−0.224	2.799	1.400	Tumor < Peritumor
L-valine biosynthesis|*Streptococcus_infantis*	0.009	−0.219	5.869	2.856	Tumor < Peritumor

### 3.5 Gastric mucosal microbiota characterization of AGC patients by kaiju

To further characterize the gastric mucosal microbiota in patients with AGC, including fungi and viruses, Kaiju annotation was used across all 177 samples, of which 87 were paired (tumor and peritumoral mucosa).

Wilcoxon signed-rank tests were used to compare microbial composition between tumor and peritumoral tissues. At the kingdom level, 16 bacteria, 23 eukaryota, and six viruses were identified. Among these, nine bacterial species, 22 eukaryotic species, and four viral species were significantly enriched in the tumor mucosa (*p* < 0.05; Supplementary Table S6A). At the phylum level, 74 viruses, three archaea, 47 bacteria, and 23 eukaryota (including eight fungi) were detected. All 20 significantly different viruses, three archaea, 14 bacteria, and 22 eukaryota (including eight fungi) were significantly abundant in tumor mucosa (*p* < 0.05, Supplementary Figure S4; Supplementary Table S6B). At the genus level, 891 genera were identified, comprising 74 viruses, seven archaea, 664 bacteria, and 144 eukaryota. Among the 281 significantly differentially abundant genera, only four bacterial genera were enriched in peritumoral mucosa, the remaining 277 genera including 20 viruses, four archaea, 156 bacteria, and 95 eukaryota (including 82 fungi) were significantly enriched in tumor mucosa (Supplementary Table S6C). At the species level, 2,662 species including 74 viruses, 12 archaea, 2,429 bacteria, and 145 eukaryota (including 82 fungi) were identified. Among the 485 significantly differentially abundant species, 13 bacteria including *Helicobacter acinonychis*, *H. cetorum*, *H. felis*, *H. pullorum*, *H. pylori*, *Lactobacillus reuteri*, and *Neisseria lactamica* were significantly enriched in peritumoral mucosa,. In contrast, the other 472 species including 20 viruses, nine archaea, 357 bacteria, and 84 eukaryota (including 42 fungi) were significantly enriched in tumor mucosa (Supplementary Table S6D).

Wilcoxon signed-rank tests further revealed: (i) 47 bacterial phyla, with 13 (e.g., Bacteroidetes, Cyanobacteria, Chlamydiae, Planctomycetes, Actinobacteria, Firmicutes) all enriched in tumor mucosa; (ii) 664 bacteria genera, of which four (*Helicobacter*, *Moraxella*, *Sulfurospirillum*, *Wolinella*) were enriched in peritumoral mucosa, while 156 others (e.g., *Enterococcus*, *Lactobacillus*, *Lactococcus*, *Streptococcus*, *Clostridium*, *Butyrivibrio*, *Clostridioides*, *Faecalibacterium*, *Ruminococcus*, *Tissierella* and etc.) were enriched in tumor mucosa; and (iii) 2,429 bacterial species, with 13 (e.g., *Neisseria lactamica*, *Xanthomonas citri*, *Campylobacter sputorum*, *Helicobacter pylori*, *H. acinonychis*, *H. cetorum*, *H. felis*, *H. pullorum*, *Wolinella succinogenes*, *Enterococcus hirae*, and *Lactobacillus reuteri*) enriched in peritumoal mucosa, and the remaining 357 species (*Selenomonas* sp. oral taxons, *Selenomonas* sp., *Lachnoanaerobaculum* sp., *Streptococcus* sp., *Lactobacillus* sp., *Campylobacter* sp., *Vibrio* sp., *Pseudomonas* sp., *Acinetobacter* sp., *Serratia* sp., *Prevotella* sp., *Enterobacter* sp.) significantly enriched in tumor mucosa.

We systematically compared the bacterial profiles obtained from MetaPhlAn4 and Kaiju across three dimensions: beta diversity patterns, abundance correlations, and differential abundance trends. Beta diversity analyses revealed significant separation between tumor and peritumoral mucosa at both genus and species levels using either method (Figure 1A; Supplementary Figure S4). Abundance correlations of the bacterial taxa between the two methods showed strong concordance, with taxa annotated by both methods aligning along the diagonal line (Supplementary Figures S5–S7). Differential abundance trends consistently demonstrated that the majority of significantly altered bacterial phyla, genera and species were enriched in tumor mucosa. Together, these results indicate that Kaiju-based annotations are highly consistent with MetaPhlAn4-based results in terms of bacterial beta diversity, abundance correlations, and differential abundance patterns.

### 3.6 PERMANOVA of phenotypes on gastric mycobiota and differential analysis

**Figure 6:**
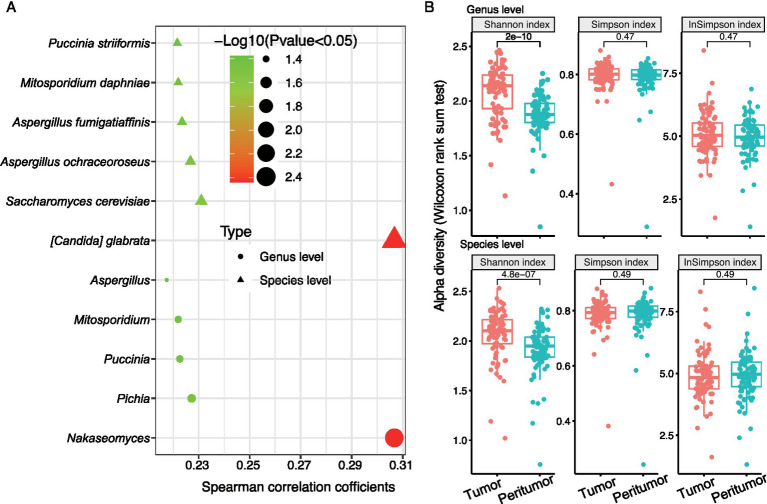
Differential analysis and PERMANOVA of phenotypes on the gastric mucosal mycobiota. **(A)** Fungal genera and species that were significantly correlated with age in peritumoral mucosa. **(B)** Fungal alpha diversity evaluated via Shannon, Simpson and inverse Simpson indices at both species and genus levels.

As described, age and tumor location significantly influenced gastric mucosal microbiota at the species and genera levels within tumoral tissues, respectively. PERMANOVA further revealed that age had a significant effect on the gastric fungal composition in peritumoral mucosa (Table 3). The results indicated that genera such as *Nakaseomyces*, *Pichia*, *Puccinia*, *Mitosporidium*, *Aspergillus*, *[Candida] glabrata*, as well as species including *Saccharomyces cerevisiae*, *Aspergillus ochraceoroseus*, *Aspergillus fumigatiaffinis*, *Mitosporidium daphniae*, *Puccinia striiformis* were significantly positively correlated with age (Figure 6A).

**Table 3:** PERMANOVA of phenotypes on fungal specie in tumor and peritumoral mucosa.

	Peritumoral mucosa			Tumoral mucosa		
	F. Model	*R* ^2^	*p* value	F. Model	*R* ^2^	*p* value
Gender	1.330	0.015	0.247	0.195	0.002	0.888
Age	2.773	0.031	**0.040**	0.721	0.008	0.478
BMI	1.062	0.012	0.317	0.445	0.005	0.607
Chemotherapy	0.576	0.007	0.714	0.357	0.004	0.742
Tumor location	1.121	0.026	0.318	0.674	0.016	0.579
Differentiated degree	0.516	0.018	0.902	0.348	0.012	0.928
lauren types	0.681	0.024	0.787	0.379	0.013	0.923
Tumor stage	1.941	0.044	0.051	0.434	0.010	0.802
Surgical resection method	0.889	0.020	0.479	0.816	0.019	0.491
Hp infection	1.025	0.012	0.339	2.239	0.025	0.109
ECOG. CCI	1.836	0.021	0.127	1.406	0.016	0.226
Nationality	1.652	0.056	0.174	1.274	0.044	0.255

Alpha diversity, assessed using Shannon index, revealed a significant reduction in fungal richness and diversity in the peritumoral mucosa (Figure 6B). To further elucidate the differences in gastric mycobiota between tumoral and peritumoral mucosa, Wilcoxon signed-rank test was applied. Totally eight fungal phyla were detected across all samples by Wilcoxon signed-rank test, including Ascomycota, Basidiomycota, Blastocladiomycota, Chytridiomycota, Cryptomycota, Mucoromycota, Microsporidia, and Zoopagomycota (Supplementary Table S6F). Notably, the abundances of all these phyla were remarkably higher in the tumoral mucosa (*p* < 0.001).

**Figure 7:**
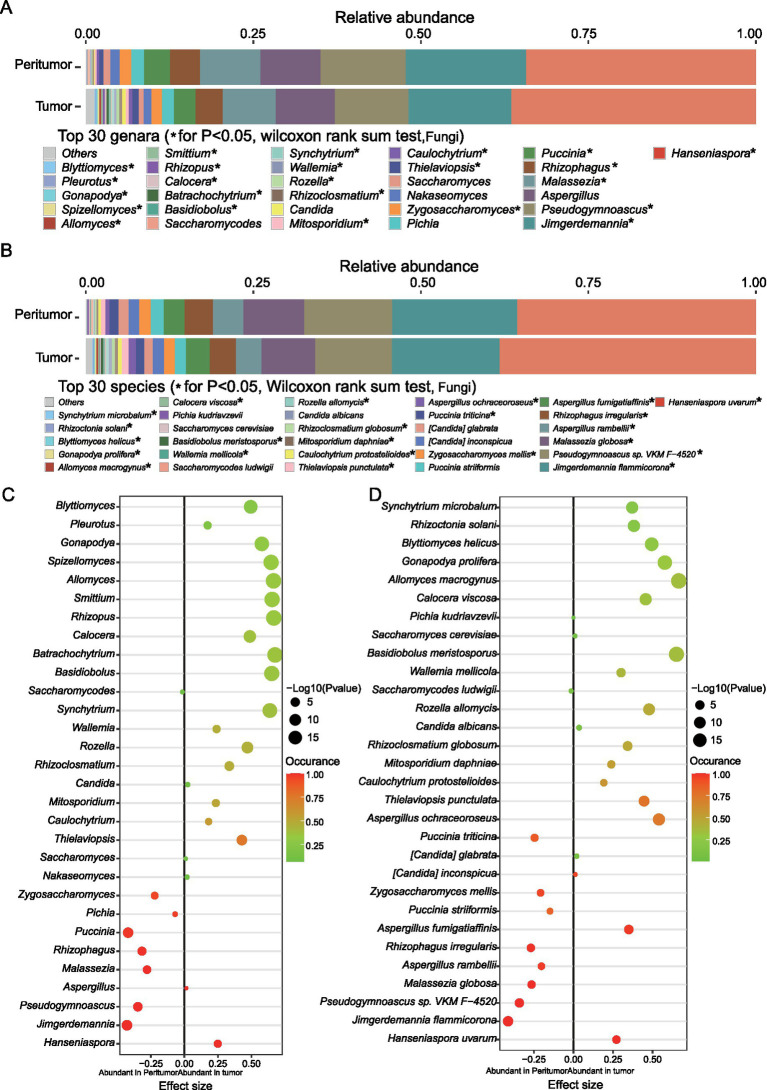
Characterization of the gastric mucosal mycobiota. Relative abundances of the top 30 most abundant fungal genera **(A)** and species **(B)**. Effect size and enrichment of the top 30 most abundant fungal genera **(C)** and species **(D)**.

At the fungal genus level, 82 genera were identified (Supplementary Table S6F). Of these, 44 genera, such as *Hanseniaspora*, *Thielaviopsis*, *Caulochytrium*, *Mitosporidium*, and *Rhizoclosmatium*, were significantly enriched in tumoral mucosa. Conversely, six genera, including *Jimgerdemannia*, *Pseudogymnoascus*, *Malassezia*, *Rhizophagus*, *Puccinia*, and *Zygosaccharomyces*, exhibited significant enrichment in peritumoral mucosa (Supplementary Figure S8). Differential analysis of the top 30 most abundant genera revealed that 17 genera, led by *Hanseniaspora*, were significantly more abundant in tumoral mucosa, whereas six genera, including *Jimgerdemannia* and *Pseudogymnoascus*, were markedly enriched in peritumoral mucosa (Figures 7A,C).

At the species level, 82 species were identified (Supplementary Table S6F). Among these, 36 species—such as *Hanseniaspora uvarum*, *Aspergillus fumigatiaffinis*, *Aspergillus ochraceoroseus*, *Thielaviopsis punctulata*, *Caulochytrium protostelioides*—were significantly enriched in gastric cancer mucosa. In contrast, seven species—*Jimgerdemannia flammicorona*, *Pseudogymnoascus* sp. VKM F-4520 (FW-2644), *Malassezia globosa*, *Aspergillus rambellii*, *Rhizophagus irregularis*, *Zygosaccharomyces mellis*, *Puccinia triticina*—showed significant depletion in peritumoral mucosa (Supplementary Figure S8). Analysis of the top 30 abundant species demonstrated that 16 species, including *Hanseniaspora uvarum* and *Aspergillus fumigatiaffinis*, were preferentially enriched in tumoral mucosa, while seven species, such as *Jimgerdemannia flammicorona*, were more abundant in peritumoral mucosa (Figures 7B,D).

### 3.7 Interactions between bacteria and fungi in AGC patients

**Figure 8:**
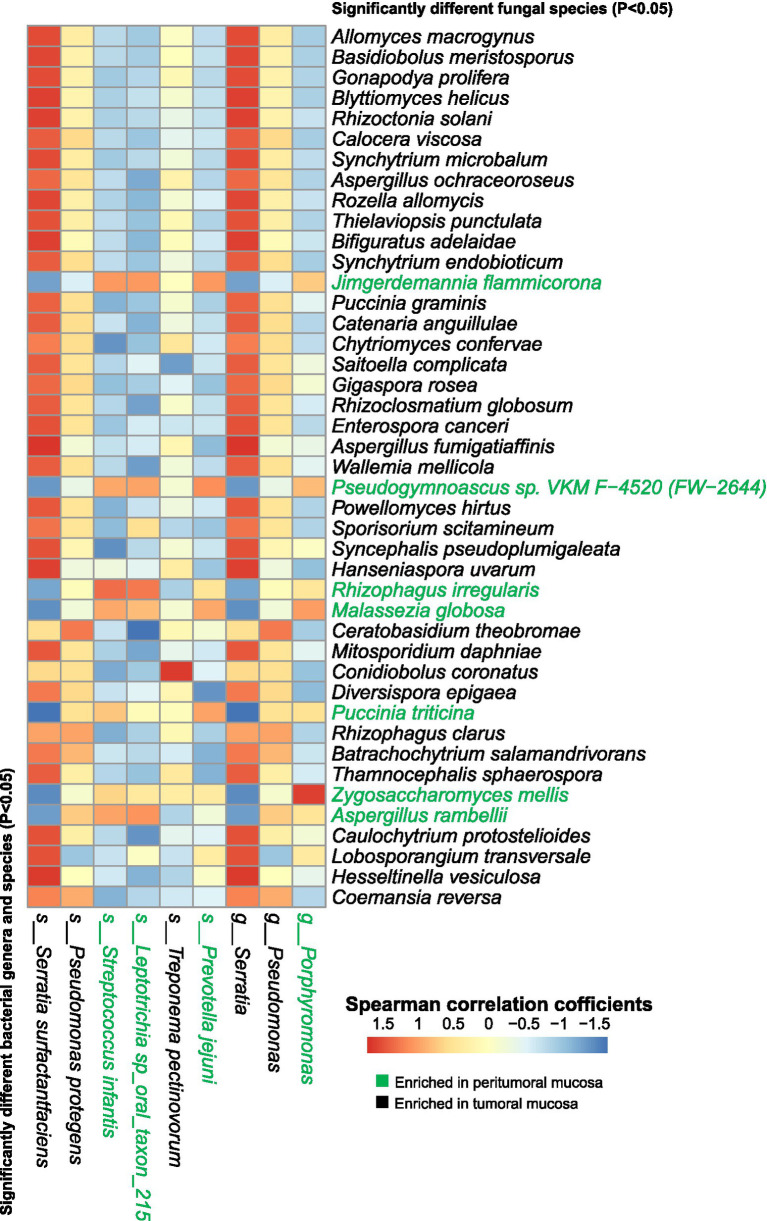
Correlation analysis between the significantly differentially abundant bacterial species annotated by MetaPhlAn4 and the significantly different fungal species annotated by Kaiju between tumor and peritumoral mucosa.

We then systematically analyzed the correlations between significantly differentially abundant bacterial and fungal taxa in AGC patients at both genus and species levels (Supplementary Figure S9, genus level; Figure 8, species level).

In tumoral mucosa, bacterial taxa such as *Pseudomonas*, *Serratia*, *Pseudomonas protegens*, *Serratia surfactantfaciens*, and *Treponema pectinovorum* were positively associated with tumor mucosa-enriched fungi (e.g., *Hanseniaspora uvarum*, *Aspergillus fumigatiaffinis*). Conversely, these bacteria were negatively correlated with peritumoral mucosa-enriched fungi, including *Jimgerdemannia flammicorona*, *Pseudogymnoascus* sp. *VKM F-4520 (FW-2644)*, *Malassezia globosa*, *Aspergillus rambellii*, *Rhizophagus irregularis*, *Zygosaccharomyces mellis*, and *Puccinia triticina*.

In peritumoral mucosa, enriched bacterial genera and species (e.g., *Porphyromonas*, *Prevotella jejuni*, *Streptococcus infantis*, *Leptotrichia sp_oral_taxon_215*) exhibited positive associations with peritumoral mucosa-enriched fungal species but negative correlations with tumor mucosa-enriched fungi. These findings highlight the complex ecological interactions between bacterial and fungal microbiomes in AGC, providing novel insights into the microbial pathogenesis of gastric cancer.

## 4 Discussion

**Figure 9:**
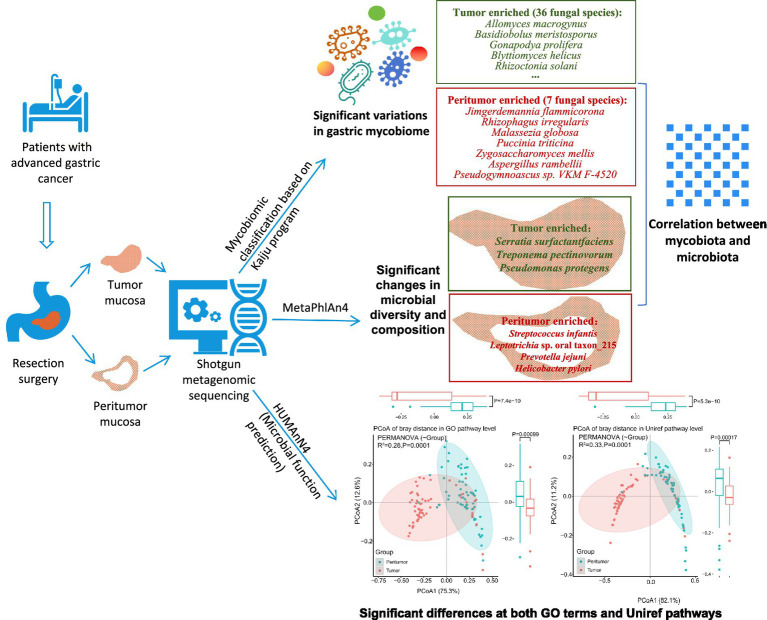
A total of 177 mucosal samples (88 peritumoral and 89 tumoral) from 91 patients with advanced gastric cancer in Northwest China were subjected to shotgun metagenomic sequencing to characterize the gastric microbiota, including both bacteria and fungi.

This study systematically investigated the compositional profiles of gastric mucosal microbiota including bacteria, fungi, and viruses, as well as functional changes of gastric mucosal bacteria in AGC patients from Northwest China, a region where high-salt dietary patterns are linked to GC risk. By analyzing microbial communities in tumoral and peritumoral tissues, we aimed to uncover microbiome-mediated carcinogenic mechanisms and identify potential diagnostic biomarkers (Figure 9).

The mean age of the 91 AGC patients were 58.84 ± 9.19 years, with a significant male predominance (female:male = 13:78), potentially linked to lifestyle factors such as alcohol consumption, smoking, and high-salt diet intake. A total of 88 peritumoral and 89 tumoral mucosal samples were collected for gastric mucosal microbiota sequencing and analysis. Taxonomic annotation via Kaiju revealed that bacteria, fungi, and two viral kingdoms (Pararnavirae and Bamfordvirae) constituted the four most abundant microbial domains in AGC patients. Pararnavirae and Bamfordvirae were significantly enriched in tumoral mucosa, suggesting their potential oncogenic roles (Niedzwiedzka-Rystwej et al., 2020). Fungal microbiota (mycobiota), comprising a small but immunologically active component of the human microbiome, have emerged as a novel cancer hallmark in pan-cancer analyses (Dohlman et al., 2022). While mycobiota dysbiosis has been reported in GC, prior studies included only 22 para-GC and 22 GC samples (Yang et al., 2022). Here we characterized fungal microbiota composition and differences between tumoral and peritumoral mucosa in 91 AGC patients using 88 peritumoral and 89 tumoral samples.

A total of 8 phyla, 82 genera, and 82 species were identified, fewer than those reported by Yang et al. (2022), likely due to annotation software differences (they used UNITE Release 8.2 vs. our use of Kaiju for fungal OTU taxonomical classification). Differential analysis identified 50 significantly distinct fungal genera and 43 species between tumoral and peritumoral mucosa, with 44 genera and 36 species showing higher abundance in tumoral mucosa. The top five fungal genera in AGC mucosal samples were *Hanseniaspora*, *Jimgerdemannia*, *Pseudogymnoascus*, *Aspergillus*, and *Malassezia*, among which *Aspergillus* has been implicated as a lethal fungus (Wang Y. et al., 2024). While *Aspergillus*, *Malassezia*, *Zygosaccharomyces*, *Thielaviopsis*, *Candida*, and *Rhodotorula* are associated with human diseases (Wang Y. et al., 2024), we found *Malassezia* and *Zygosaccharomyces* enriched in peritumoral mucosa, whereas *Thielaviopsis* was tumor-enriched. *Candida albicans*, previously linked to GC, showed no significant difference between tumoral and peritumoral mucosa here. Tumoral mucosa was significantly enriched for *Hanseniaspora uvarum* and *Aspergillus fumigatiaffinis*, while *Jimgerdemannia flammicorona*, *Malassezia globosa*, *Aspergillus rambellii*, and *Rhizophagus irregularis* were peritumoral-enriched. *J. flammicorona* is a putative ectomycorrhizal fungus associated with Pinaceae species but unreported in humans (Desiro et al., 2017); *M. globosa* is a skin microecosystem resident with opportunistic pathogenic potential; *A. rambellii* is pathogenic and linked to colorectal cancer (Lin et al., 2022); and *R. irregularis* is a model arbuscular mycorrhizal fungus (Kokkoris et al., 2024). In all, we revealed the compositional characteristics and inter-tissue differences of gastric mucosal mycobiota in AGC patients, uncovering previously unreported fungal genera and species.

Then we primarily investigated the mucosal bacterial composition and functional profiles in AGC patients. Twelve bacterial phyla were identified, with Proteobacteria, Firmicutes, and Bacteroidetes comprising the dominant phyla; however, no significant intergroup differences were observed among these major phyla. Totally 98 bacterial genera and 278 species were detected in the gastric mucosa of AGC patients. Notably, the genus *Serratia*, known for producing bio-active secondary metabolites such as prodigiosin, serrawettins and biosurfactants (Clements et al., 2019), was significantly enriched in tumorous mucosa. *S. surfactantfaciens* has been reported to synthesize prodigiosin and serrawettin W2 (Su et al., 2016). Similarly, *P. protegens*, a gram-negative bacterium producing antimicrobial, insecticidal, and plant growth-promoting secondary metabolites (Garrido-Sanz et al., 2023), exhibited significant enrichment in tumoral mucosa. *T. pectinovorum*, isolated from periodontitis patients (Wyss et al., 2001), was found to produce lipopolysaccharides (Kesavalu et al., 2002) and induce robust macrophage chemotactic protein 1 (MCP-1) production in human gingival fibroblasts (Nixon et al., 2000), was enriched in AGC tumoral mucosa. Although Hp is a main risk factor for GC (Wang et al., 2014), prior studies (Wang G. et al., 2024), consistent with our findings, have shown reduced Hp abundance in tumoral mucosa compared to peritumoral mucosa (Dai et al., 2021). *Prevotella jejuni*, originally isolated from the small intestine of a celiac disease child (Hedberg et al., 2013), was significantly increased in peritumoral mucosa. *Leptotrichia* species, non-motile facultative anaerobic bacteria primarily found in the oral cavity, intestines, and urogenital tract, which ferment carbohydrates to produce organic acids (e.g., lactic acid) and may cause opportunistic bacteremia in neutropenic patients (Eribe and Olsen, 2017), were increased in peritumoral mucosa. *Streptococcus*, previously linked to GC tumoral tissues (Dai et al., 2021), and *Streptococcus infantis* a breast cancer-associated bacterium (Xie et al., 2022), were significantly enriched in peritumoral mucosa of AGC patients. Collectively, our study revealed distinct gastric mucosal microbiota profiles in tumoral and peritumoral mucosa of AGC patients, identifying enrichment of beneficial species (e.g., *S. surfactantfaciens*, *P. protegens*) and pathogenic bacteria (e.g., *T. pectinovorum*) in tumoral mucosa, alongside infectious taxa (e.g., *Porphyromonas*, *Leptotrichia* sp. oral_taxon_215, *S. infantis*, *P. jejuni*, *Hp*) in peritumoral mucosa.

Predictive functional profiling of mucosal bacteria revealed enrichment of substrates and energy metabolism pathways in tumoral mucosa, including D-fructuronate degradation, glucose/glucose-1-phosphate degradation, D-galacturonate degradation, palmitate biosynthesis, superpathway of thiamine diphosphate biosynthesis I, fatty acid biosynthesis/*β*-oxidation, superpathway of histidine/purine/pyrimidine biosynthesis, mitochondrial NADPH production (yeast), ketogluconate metabolism and etc. These pathways were mainly mediated by *Serratia* spp., which are notable nosocomial pathogens (Hejazi and Falkiner, 1997), with increased abundance in tumoral mucosa. Cancer cells typically rewire metabolism to sustain ATP and macromolecules production for proliferation and survival (Koundouros and Poulogiannis, 2020). GC cells exhibit “Warburg effect”-like metabolic reprogramming, characterized by enhanced glycolysis and lactate production (Zheng et al., 2023). Fatty acid metabolism, critical for membrane structure and energy production (Koundouros and Poulogiannis, 2020), may support tumor cell survival and proliferation via microbial activity in tumoral mucosa. In contrast, peritumoral mucosal functions were enriched in L-tryptophan biosynthesis, pyrimidine deoxyribonucleotide phosphorylation, molybdopterin biosynthesis, TCA cycle, and peptidoglycan biosynthesis, primarily mediated by *S. infantis* and *Haemophilus* spp. (enriched in peritumoral mucosa). L-tryptophan (Trp) metabolism influences digestive tumor progression by modulating immunosuppressive microenvironments (Badawy, 2022; Liu and Zhai, 2021; Yu et al., 2024), with increased biosynthesis potentially reflecting pre-malignant transformation in adjacent tissues. Elevated TCA cycle intermediates (cis-aconitate, *α*-ketoglutarate, fumarate) in GC plasma (Kim et al., 2022), further link microbial metabolism to tumor biology.

However, as noted by Fierer et al. (2025), negative controls should be considered in metagenomic sequencing. Regrettably, we did not include such controls in our libarary construction and sequencing due to the very low total DNA content. Although we could not include true negative controls due to external sequencing logistics, the application of statistical *decontam*ination tools—*decontam*—strengthens the validity of the observed biological signals. In our upcoming multi-center sampling study, we plan to include negative controls and further optimize the procedures for micro-library construction and sequencing.

In conclusion, our study systematically characterized the community profiling and intergroup differences of the mucosal microbiota and analyzed bacterial functional predictive profiles in tumoral and peritumoral mucosa of AGC patients from Northwest China. By revealing microbial metabolic reprogramming patterns in both tumoral and peritumoral microenvironments, this research establishes a foundational framework for subsequent microbiome-targeted studies on the role of mucosal microbiota in GC pathogenesis and progression. These findings hold significant promise for driving innovations in precision medicine strategies and region-specific GC prevention and control initiatives.

## Data Availability

The datasets presented in this study can be found in online repositories. The names of the repository/repositories and accession number(s) can be found at: https://www.ncbi.nlm.nih.gov/, PRJNA1067082.

## Glossary

GC: Gastric cancer
AGC: Advanced gastric cancer
CTP: Cytidine triphosphate
TCA: Tricarboxylic acid cycle
VEGF: Vascular endothelial growth factor
Hp: *Helicobacter pylori*
MetaPhlAn: Metagenomic Phylogenetic Analysis
HUMAnN: HMP Unified Metabolic Analysis Network
DNA: Deoxyribonucleic acid
RNA: Ribonucleic Acid
PE: Pair-end
GO: Gene Ontology
BP: Biological process
MF: Molecular function
CC: Cellular component
UniRef: The UniProt Reference Clusters
PCoA: Principal Coordinate Analysis
PERMANOVA: Permutational multivariate analysis of variance
BMI: Body mass index
OTU: Operational Taxonomic Unit
NADPH: Nicotinamide Adenine Dinucleotide Phosphate
Trp: L-tryptophan
ATP: Adenosine Triphosphate
