# Supplementary material for: Characterization of the gastric mucosal microbiota in tumoral and peritumoral mucosa in patients with advanced gastric cancer from Northwest China
Source: Front Microbiol. 2026 Jun 17;17:1763714. doi: 10.3389/fmicb.2026.1763714 (PMC13319720; doi:10.3389/fmicb.2026.1763714)
Supplement: Supplementary file 1 [file supplementary_file_1.zip › Supplementary Figures S1-S9.PDF]

Supplementary figures

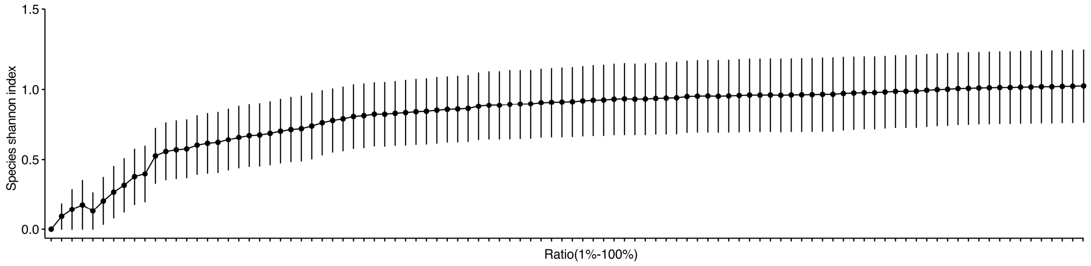

Figure S1. Rarefraction curves of randomly selected eight samples.

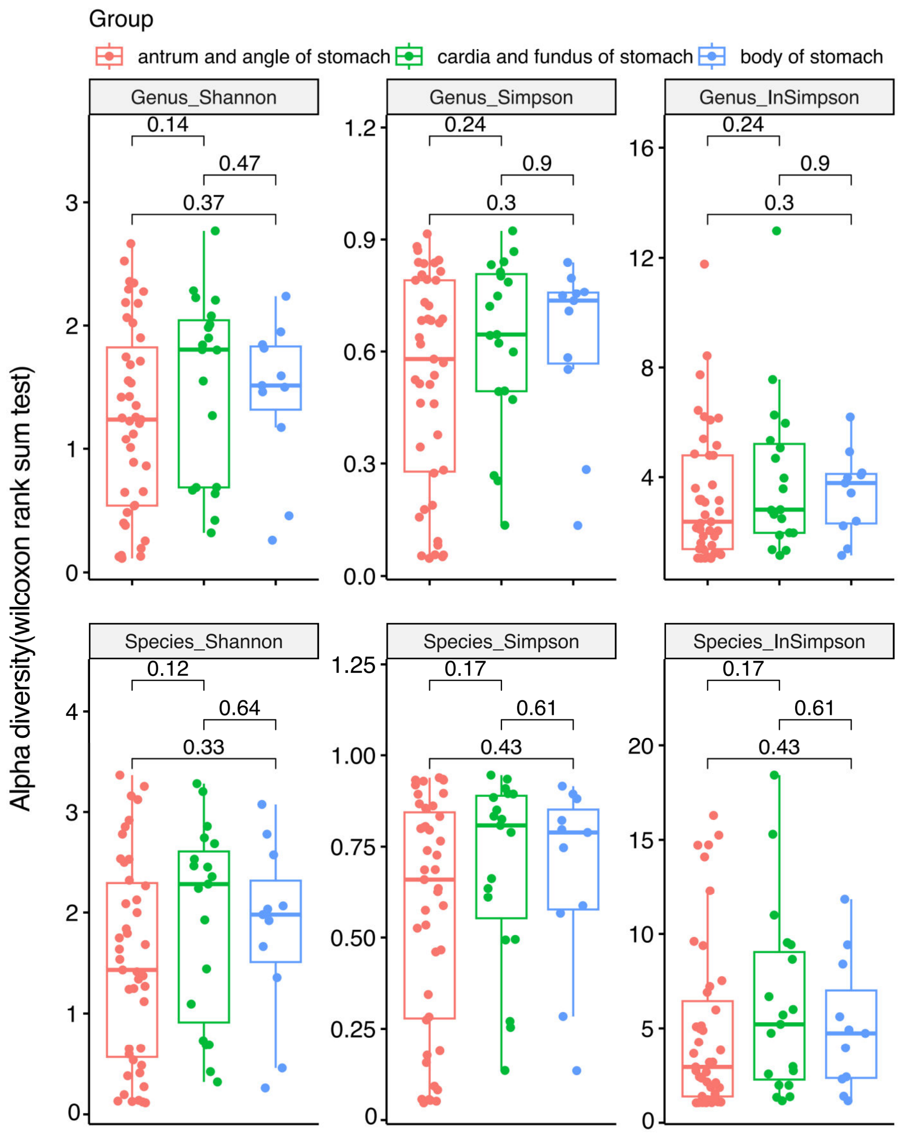

Figure S2. Alpha diversity of bacterial genera and species across three different tumor locations.

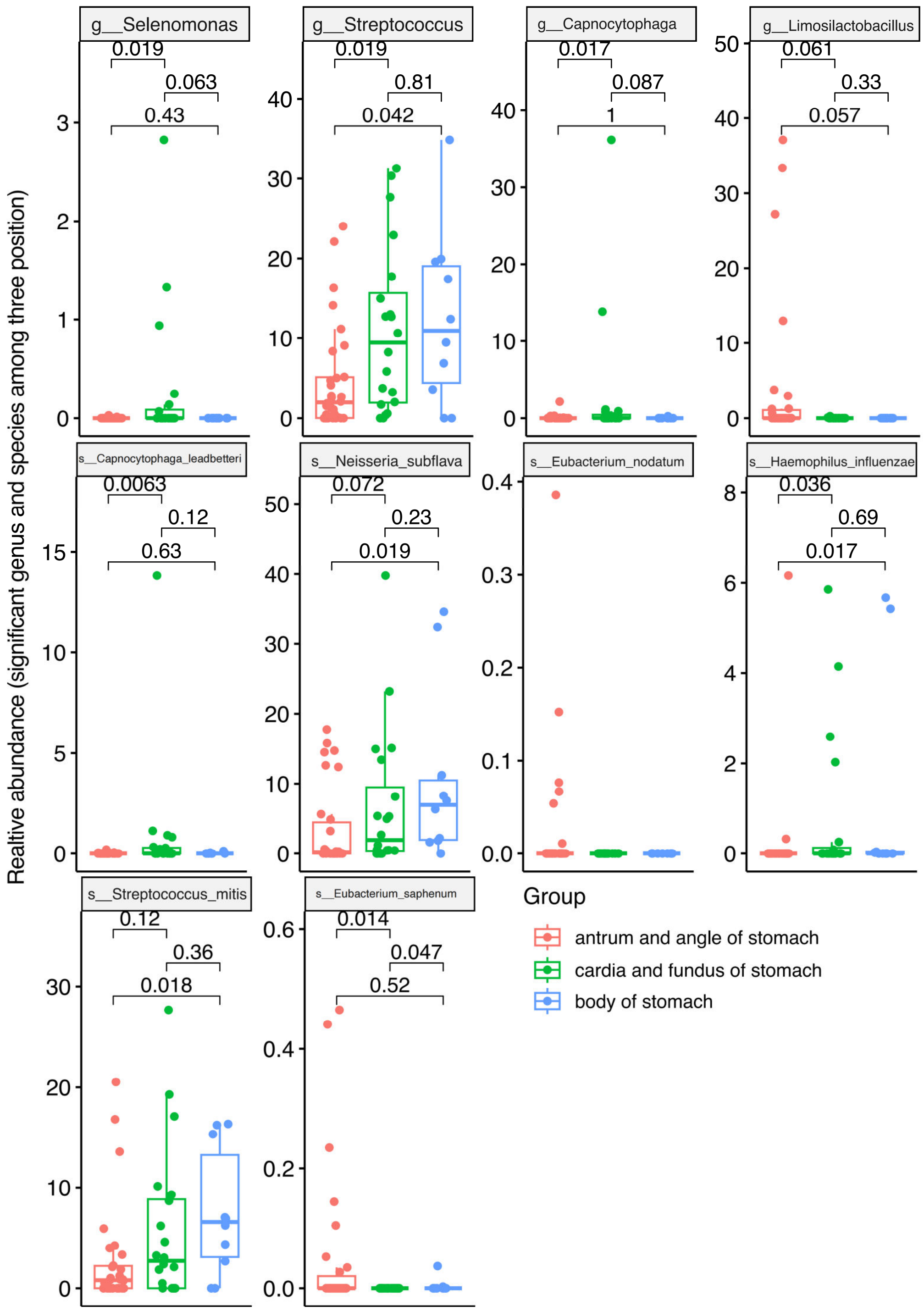

Figure S3. Intergroup differences in four markedly differential genera and six significantly distinct species across three tumor locations.

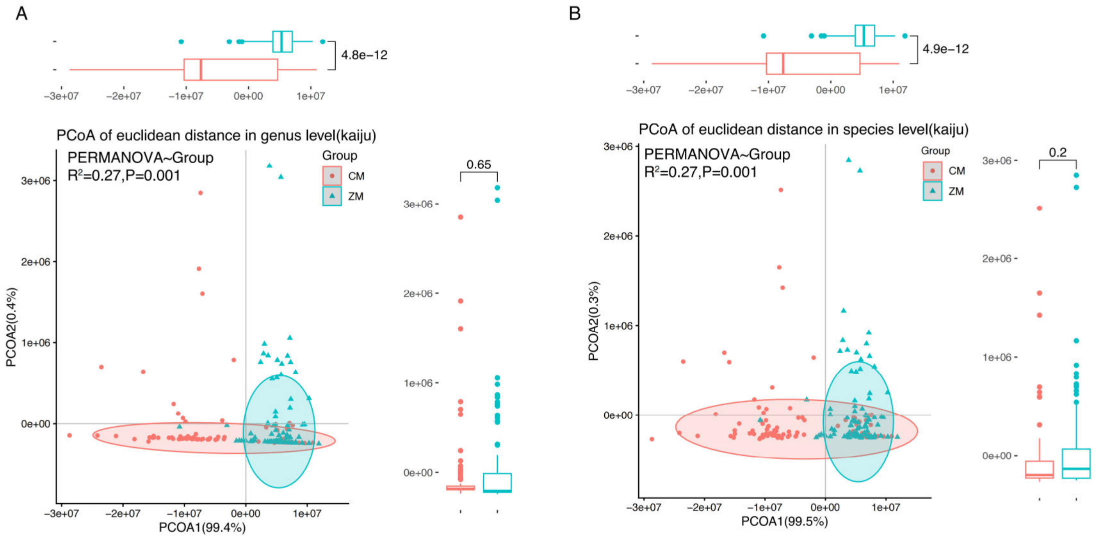

Figure S4. Alpha diversity of the bacterial genera and species annotated by Kaiju.

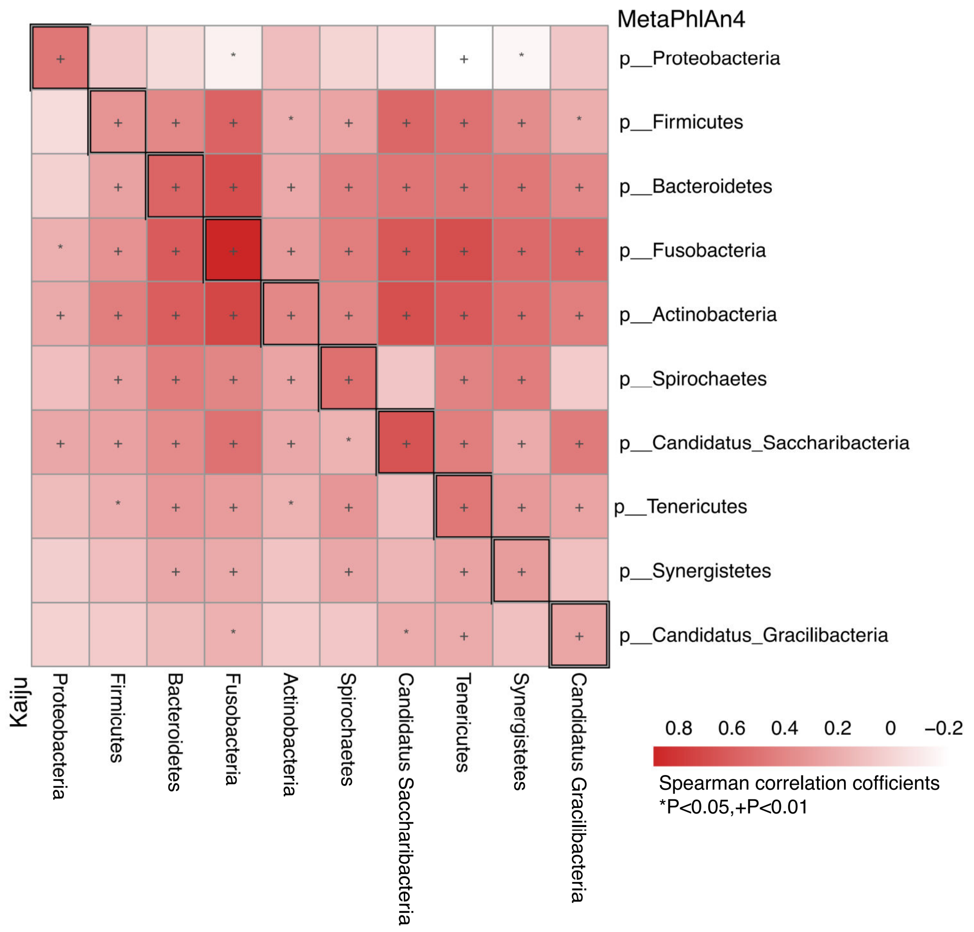

Figure S5. Correlation analysis of bacterial phyla annotated by Kaiju and Metaphlan4.

Figure S6. Correlation analysis of bacterial genera annotated by Kaiju and Metaphlan4.

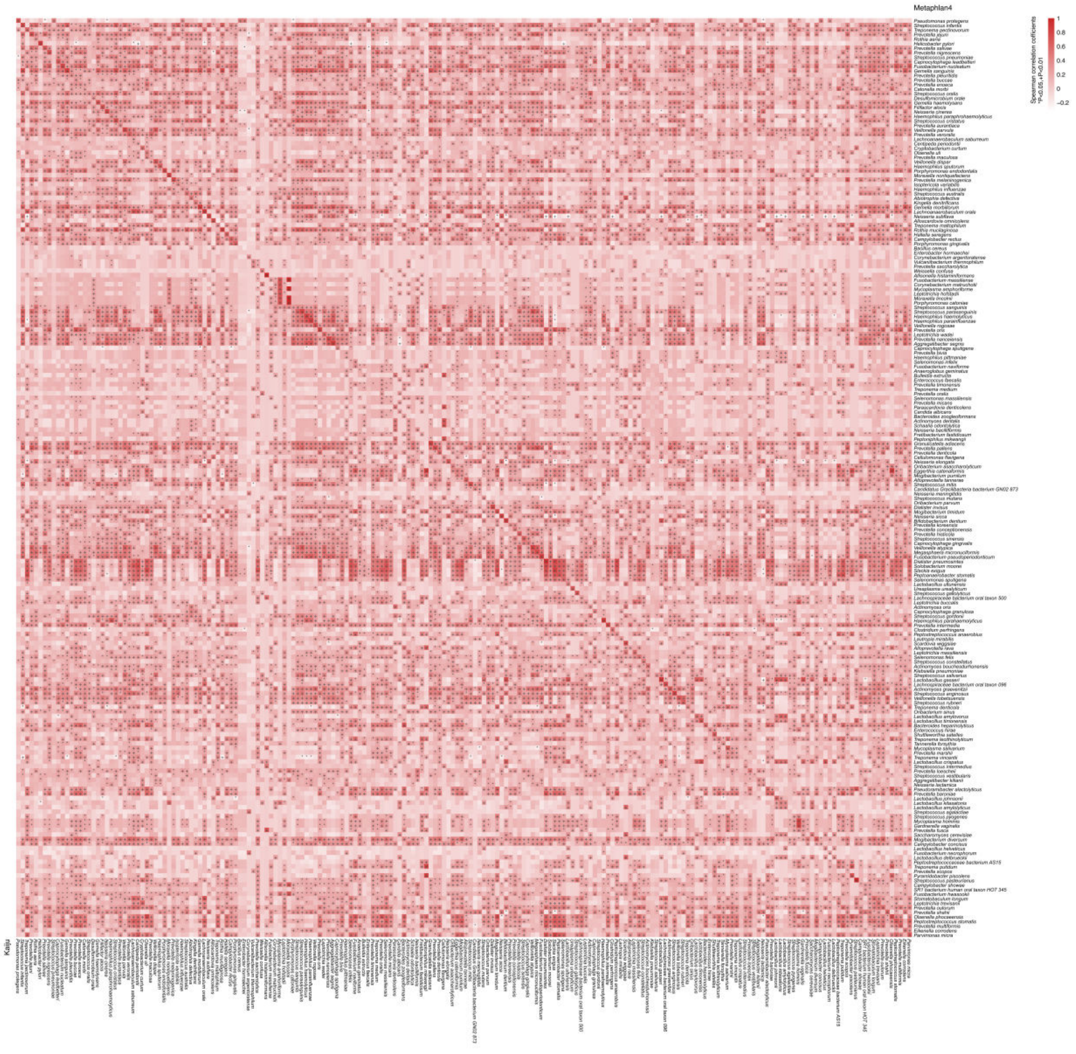

Figure S7. Correlations of bacterial species annotated by Kaiju and Metaphlan4.

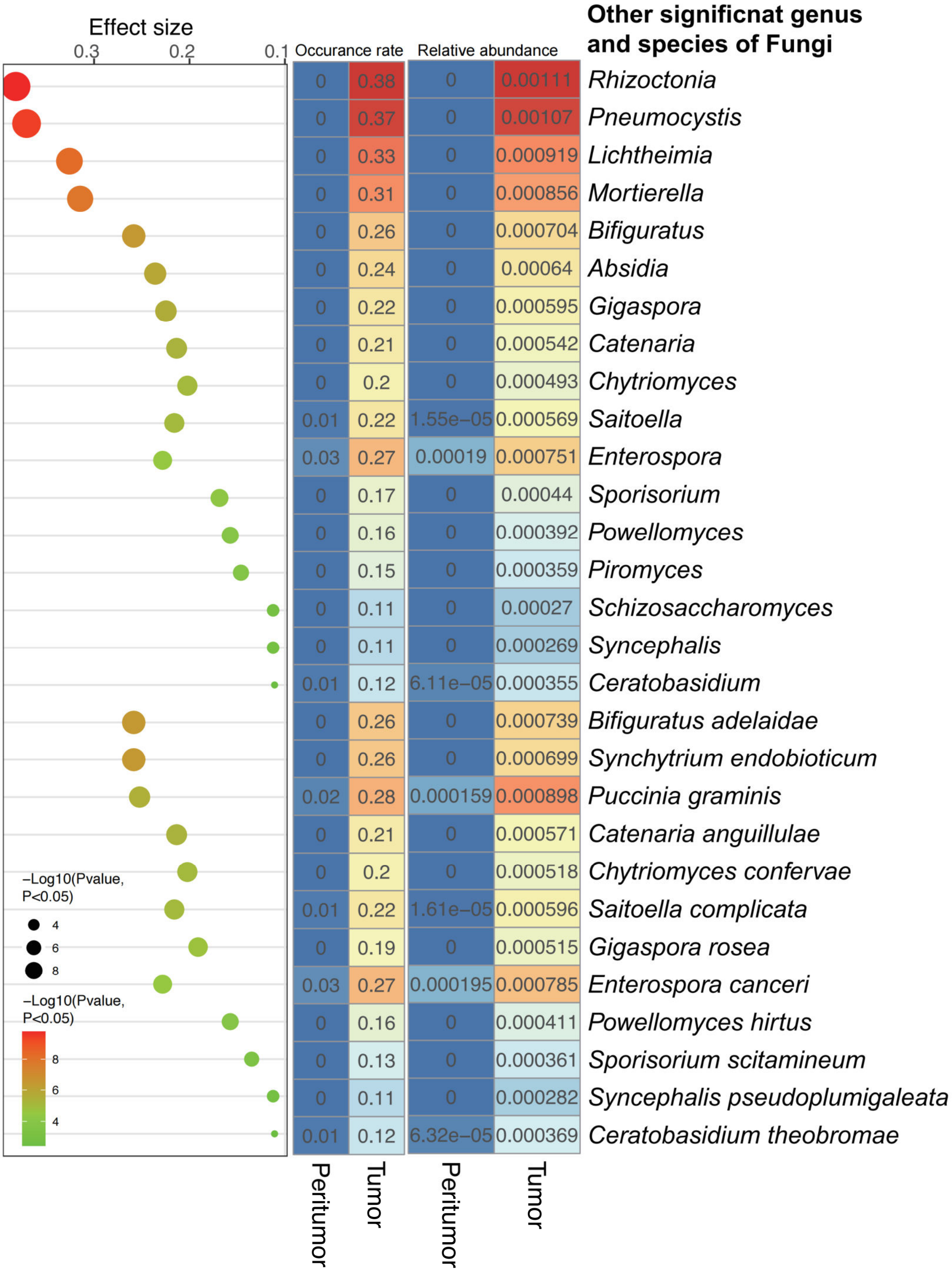

	Occurance rate Peritumor	Occurance rate Tumor	Relative abundance Peritumor	Relative abundance Tumor
Rhizoctonia	0	0.38	0	0.00111
Pneumocystis	0	0.37	0	0.00107
Lichtheimia	0	0.33	0	0.000919
Mortierella	0	0.31	0	0.000856
Bifiguratus	0	0.26	0	0.000704
Absidia	0	0.24	0	0.00064
Gigaspora	0	0.22	0	0.000595
Catenaria	0	0.21	0	0.000542
Chytriomyces	0	0.2	0	0.000493
Saitoella	0.01	0.22	1.55e−05	0.000569
Enterospora	0.03	0.27	0.00019	0.000751
Sporisorium	0	0.17	0	0.00044
Powellomyces	0	0.16	0	0.000392
Piromyces	0	0.15	0	0.000359
Schizosaccharomyces	0	0.11	0	0.00027
Syncephalis	0	0.11	0	0.000269
Ceratobasidium	0.01	0.12	6.11e−05	0.000355
Bifiguratus adelaidae	0	0.26	0	0.000739
Synchytrium endobioticum	0	0.26	0	0.000699
Puccinia graminis	0.02	0.28	0.000159	0.000898
Catenaria anguillulae	0	0.21	0	0.000571
Chytriomyces confervae	0	0.2	0	0.000518
Saitoella complicata	0.01	0.22	1.61e−05	0.000596
Gigaspora rosea	0	0.19	0	0.000515
Enterospora canceri	0.03	0.27	0.000195	0.000785
Powellomyces hirtus	0	0.16	0	0.000411
Sporisorium scitamineum	0	0.13	0	0.000361
Syncephalis pseudoplumigaleata	0	0.11	0	0.000282
Ceratobasidium theobromae	0.01	0.12	6.32e−05	0.000369

Figure S8. Other significantly different fungal genera and species between tumor and peritumoral mucosa.

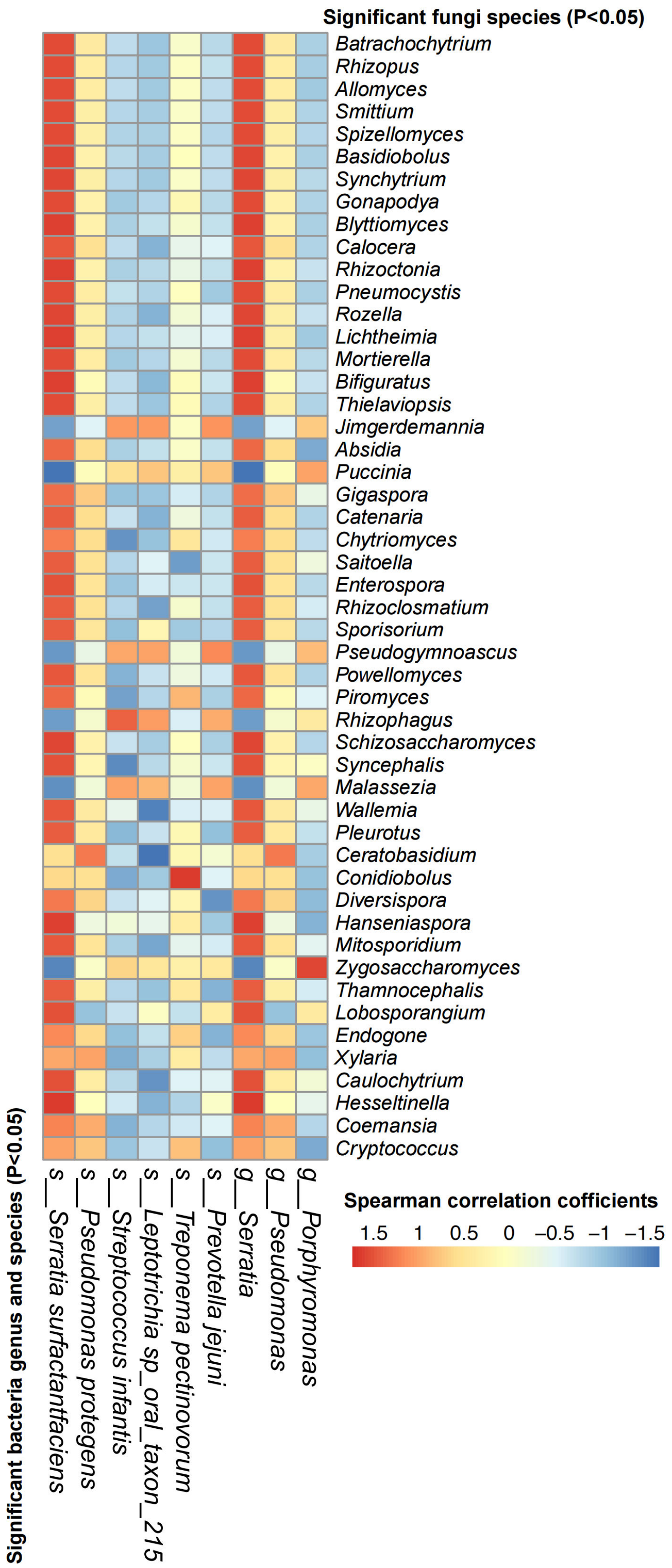

Figure S9. Correlation analysis between significantly different bacterial genera/species annotated by MetaPhlAn4 and fungal genera annotated by Kaiju.
